# The Influence of Orthopedic Surgery on Circulating Metabolite Levels, and their Associations with the Incidence of Postoperative Delirium

**DOI:** 10.3390/metabo12070616

**Published:** 2022-07-01

**Authors:** Mijin Jung, Xiaobei Pan, Emma L. Cunningham, Anthony P. Passmore, Bernadette McGuinness, Daniel F. McAuley, David Beverland, Seamus O’Brien, Tim Mawhinney, Jonathan M. Schott, Henrik Zetterberg, Brian D. Green

**Affiliations:** 1 Institute for Global Food Security, School of Biological Sciences, Queen’s University Belfast, 8 Malone Road, Belfast BT9 5BN, Northern Ireland, UK; mjung01@qub.ac.uk (M.J.); x.pan@qub.ac.uk (X.P.); 2 Centre for Public Health, Institute of Clinical Sciences, Queen’s University Belfast, Block B, Royal Victoria Hospital Site, Grosvenor Road, Belfast BT12 6BA, Northern Ireland, UK; emma.cunningham@qub.ac.uk (E.L.C.); p.passmore@qub.ac.uk (A.P.P.); b.mcguinness@qub.ac.uk (B.M.); 3 Centre for Experimental Medicine, Wellcome-Wolfson Institute for Experimental Medicine, Queen’s University Belfast, 97 Lisburn Road, Belfast BT9 7BL, Northern Ireland, UK; d.f.mcauley@qub.ac.uk; 4 Outcomes Assessment Unit, Musgrave Park Hospital, Belfast Trust, Stockman’s Lane, Belfast BT9 7JB, Northern Ireland, UK; david.beverland@belfasttrust.hscni.net; 5 Cardiac Surgical Intensive Care Unit, Belfast Trust, Royal Victoria Hospital, Grosvenor Road, Belfast BT12 6BA, Northern Ireland, UK; seamus.obrien@belfasttrust.hscni.net (S.O.); tim.mawhinney@belfasttrust.hscni.net (T.M.); 6 Department of Neurodegenerative Disease, UCL Institute of Neurology, Queen Square, London WC1E 6BT, UK; j.schott@ucl.ac.uk (J.M.S.); h.zetterberg@ucl.ac.uk (H.Z.); 7 UK Dementia Research Institute at UCL, London WC1E 6BT, UK; 8 Clinical Neurochemistry Laboratory, Sahlgrenska University Hospital, House V, S-431 80 Mölndal, Sweden; 9 Department of Psychiatry and Neurochemistry, Institute of Neuroscience and Physiology, The Sahlgrenska Academy at the University of Gothenburg, House V, S-431 80 Mölndal, Sweden; 10 Hong Kong Center for Neurodegenerative Diseases, Clear Water Bay, Hong Kong, China

**Keywords:** orthopedic surgery, postoperative delirium, spermine, ornithine, polyamine

## Abstract

The mechanisms underlying the occurrence of postoperative delirium development are unclear and measurement of plasma metabolites may improve understanding of its causes. Participants (*n* = 54) matched for age and gender were sampled from an observational cohort study investigating postoperative delirium. Participants were ≥65 years without a diagnosis of dementia and presented for primary elective hip or knee arthroplasty. Plasma samples collected pre- and postoperatively were grouped as either control (*n* = 26, aged: 75.8 ± 5.2) or delirium (*n* = 28, aged: 76.2 ± 5.7). Widespread changes in plasma metabolite levels occurred following surgery. The only metabolites significantly differing between corresponding control and delirium samples were ornithine and spermine. In delirium cases, ornithine was 17.6% higher preoperatively, and spermine was 12.0% higher postoperatively. Changes were not associated with various perioperative factors. In binary logistic regression modeling, these two metabolites did not confer a significantly increased risk of delirium. These findings support the hypothesis that disturbed polyamine metabolism is an underlying factor in delirium that warrants further investigation.

## 1. Introduction

Delirium is a clinical syndrome characterized by disturbances in attention, awareness, and cognition [1]. Delirium is common in older people following surgery, and incidence varies depending on the population and type of surgery [2,3]. The incidence rates following orthopedic surgery, for example, range between 4.5 and 41.2% [4,5,6]. Delirium is unpleasant for patients, families, and staff; it is associated with increased subsequent morbidity and mortality, and it incurs additional healthcare expenditure [7,8]. The pathophysiological processes underlying delirium remain poorly understood, and this hinders efforts to prevent and treat it. For new interventions to be conceived for lowering the incidence of this common, unpleasant, and potentially dangerous syndrome, we must first understand the biochemical basis for its development. Since neurodegenerative disease appears to predispose individuals to a higher risk of delirium, there is potential for wider lessons to be learned from investigating delirium. Elective orthopedic study populations make it feasible to undertake detailed preoperative and premorbid cognitive assessments. This contrasts sharply with hip fracture populations whose profiles are typically older, with more trauma and co-morbidities [9,10].

Mass spectrometry methods for profiling metabolites have been used extensively to investigate disease processes and discover novel disease biomarkers [11,12]. Key advantages of these techniques are that they are high-throughput, cover a broad range of metabolites, and can be applied to many sample types, including plasma, cerebrospinal fluid (CSF), and mammalian tissues [13,14,15]. Blood metabolite profiling is frequently undertaken as sample collection is relatively routine, and the obtained profiles reflect metabolic changes across the body [16,17].

Only a small number of studies to date have explored changes in metabolites associated with delirium. Previous work from our group has shown arginine metabolism is altered preoperatively in CSF from delirium patients [15]. Other studies have shown altered levels of five amino acids (tyrosine, phenylalanine, tryptophan, glutamine, and S-methylcysteine), three fatty acids (linolenic acid, eicosapentaenoic acid, and linoleic acid), various amines (spermidine, putrescine, and phenethylamine), and also uracil [18,19,20,21,22,23]. In delirium patients, plasma levels of glutamine, tryptophan, S-methylcysteine, fatty acids, and uracil are decreased, but levels of phenylalanine and phenylethylamine are increased [20,24]. Furthermore, increased CSF levels of glutamine, tryptophan, spermidine, and putrescine have been observed, as well as decreased levels of PEA [15,19,23]. Such metabolite changes reflect the potential alterations underlying the occurrence of delirium, including the dysregulation of neurotransmitters, hormones, and energy metabolism [20,21,25]. Given that various metabolites appear to be altered in delirium, the current investigation had a different focus. The aim was to understand the perioperative context for metabolite changes associated with delirium in an elective surgical population. To do this, metabolite profiling was undertaken in plasma samples acquired both before and after hip or knee arthroplasty [26,27]. The dataset acquired was then interrogated to understand the metabolites, which were altered by surgery, and more importantly how this differed in those patients who were predisposed to delirium. Plasma metabolites that appeared to be implicated in delirium were then assessed as potential risk factors, and their association with various perioperative factors was assessed. 

## 2. Materials and Methods

### 2.1. Participants

Participants were sampled from an observational cohort study, the methods for which are published [26]. In brief, between March 2012 and October 2014, participants aged over 65 years who were scheduled for primary elective hip or knee arthroplasty under spinal anesthesia were recruited. Exclusion criteria included a diagnosis of dementia or other neurodegenerative condition. Written informed consent was obtained from all participants, and the study was approved by local ethical committee procedures (Office for Research Ethics Committees Northern Ireland; REC reference: 10/NIR01/5). Participants had plasma sampled preoperatively, at the time of anesthetic administration, and postoperatively on Day 1. Postoperatively, participants were assessed daily for delirium as previously described [15,26,27].

Plasma samples from participants developing delirium (*n* = 28, aged: 76.2 ± 5.7) and age and sex-matched controls (*n* = 26, aged: 75.8 ± 5.2) underwent metabolomic analysis [15]. To investigate the effect of surgery on metabolites and which metabolites affect the development of delirium postoperatively, participants were divided initially into 2-way comparisons (the phase of surgery: preoperative (pre-op) and postoperative (post-op)) and then into 4-way comparison (the phase of surgery with participants’ delirium status: preoperative control (pre-ctrl), preoperative delirium (pre-del), postoperative control (post-ctrl), and postoperative delirium (post-del)) groups.

Demographic variables collected included: age, sex, type of surgery (hip or knee), years in education, Charlson Comorbidity Index (CCI), estimated IQ, Bristol activities of daily living scale (BALD) score, Vertical Visual Analog Pain Scores, and American Society Anesthesiologists (ASA) physical status [15,26,27]. Neuropsychological tests included Color Trails 2, New York university paragraph recall test, and Mini-Mental State Examination (MMSE).

Perioperative anesthetic and surgical data were collected and considered as potential confounders as below. Data available included: premedications; duration of surgery; intraoperative blood loss; intraoperative intravenous fluid volumes administered; use of peripheral nerve block/plexus blockade; general anesthetic and intraoperative sedation; perioperative analgesics and antiemetics; intraoperative tachycardia and/or hypotension; prophylactic antibiotics administered.

### 2.2. Sample Collection and Preparation

Venous blood was sampled from fasted participants into lithium heparin bottles preoperatively at the time of anesthetic administration and postoperatively on Day 1. Samples were transported to the laboratory on wet ice within 12 h of collection, where they were centrifuged at 4 °C at 3750 rpm for 10 min and 500 mL aliquots of the supernatant pipetted (Sarstedt, Germany, Catalog No. 70.762) into 1.5 mL polypropylene tubes (Sarstedt, Germany, Catalog No. 72.69.001) and stored at −80 °C.

### 2.3. Targeted Metabolomics

Quantitative mass spectrometry-based metabolomic profiling was carried out by the Biocrates Absolute IDQ p180 (BIOCRATES, Life Science AG, Innsbruck, Austria). All plasma samples were prepared according to the manufacturer’s instructions and analyzed on a triple-quadrupole mass spectrometer (Xevo TQ-MS, Waters Corporation, Milford, MA, USA). Metabolites (amino acids and biogenic amines) were derivatized using phenylisothiocyanate (PITC) in the presence of isotopically labeled internal standards, separated using a UPLC (I-Class, Waters Corporation, Wilmslow, UK) system with a reverse-phase column (Waters ACQUITY UPLC BEH C18 2.1 × 50 mm, 1.7 μm; UK) and quantified using a triple-quadrupole mass spectrometer (Xevo TQ-MS, Waters Corporation, UK) operating in the multiple reaction monitoring (MRM) mode. All the remaining metabolites (acylcarnitines, hexoses, glycerophospholipids, and sphingolipids) were quantified using the same mass spectrometer without column separation by the flow injection analysis (FIA) operating in MRM mode. Metabolite concentrations were calculated and expressed as micromole (μM). The mean of the coefficient of variation (CV) for the 184 metabolites in repeated quality controls was 0.12, and 85% of the metabolites had a CV of <0.15. Following these analyses, 138 metabolites were selected above limit of detection (LOD) values.

### 2.4. CSF Aβ42, t-tau, and p-tau Analysis

Spinal anesthesia was carried out, fasting, in the sitting or lateral positions using 25-gauge (diameter 0.53 mm; length 90 mm) Whitacre-type spinal needles with graduated metal introducers (Vygon). Once CSF was obtained, a 5 mL syringe (BD Plastipak; BD) was attached to the spinal needle, and up to 5 mL of CSF was withdrawn by the anesthetist. The CSF was immediately transferred to a 30 mL sterile polypropylene universal container (Unisurge), which was placed on wet ice in an insulated container. All samples were transported to the laboratory within 12 h of collection. CSF samples were centrifuged at 4 °C at 3000 rpm/1811× *g* for 5 min with no brake and 500 mcl aliquots of the supernatant pipetted (Sarstedt, Germany, Catalog No. 70.762) into 1.5 mL polypropylene tubes (Sarstedt, Germany, Catalog No. 72.69.001) and stored at −80 °C. 

CSF samples were transported to the Leonard Wolfson Biomarker Laboratory, University College London on dry ice. There, CSF Aβ42, t-tau, and p-tau concentrations were measured by a single, trained, technician, blinded to the clinical data, using the INNOTEST β-amyloid (1–42), hTau Ag, and Phospho-tau (181 P) ELISA kits, respectively (Fujirebio, Ghent, Belgium), run on a FLUO star Omega BMG LABTECH instrument (chemiluminescent plate reader) according to the manufacturer’s protocols. Samples were thawed at 21 °C in an air-conditioned lab and vortexed before use. CSF biomarker values were determined using Omega software (version 5.10 R2, Ontario, Canada) and a 4-parameter curve fit. Intra- and interassay CVs, respectively, were 2.0% and 3.9% for Aβ42, 3.3% and 8.1% for T-tau, and 1.6% and 5.1% for P-tau. Longitudinal stability in the measurements is further monitored in the laboratory by participation in the Alzheimer’s Association Quality Control program.

### 2.5. Statistical Analysis

MetaboAnalyst software (version 5.0, Canada) was used for the data analysis. Data were normalized to the median and autoscaled before using PLS-DA scores and VIP scores. A normality test was carried out by SPSS (version 26, Chicago, IL, USA). To compare the difference between pre and post-op metabolites, the paired Student’s t-test for parametric data and the Wilcoxon Mann-Whitney test for nonparametric data were performed. False discovery rates (FDR, q-value) were calculated based on Benjamini–Hochberg to determine if metabolites were statistically different between the two groups (pre-op and post-op, q < 0.05). For multiple comparisons on metabolites over 4 groups (pre-ctrl, pre-del, post-ctrl, and post-del), a one-way ANOVA test for parametric data and Kruskal–Wallis test for nonparametric data were conducted. PLS-DA was carried out to highlight significant metabolites, which explain the maximum amount of variation between the groups. A VIP plot was created to identify the top 15 metabolites responsible for the observed separation between groups. 

Binary logistic regression was carried out to investigate risk factors for delirium. Statistical tests that we used were independent *t*-test, χ2 analysis, and Mann-Whitney U test to compare the difference between control and delirium groups. To investigate whether perioperative changes in metabolites could be due to perioperative factors, correlation analyses were undertaken between perioperative variables and % change of metabolites, and the q-value from Benjamini–Hochberg was confirmed.

## 3. Results

### 3.1. Clinical Characteristics

Baseline characteristics of the selected participants with and without delirium are displayed in Table S1. Control and delirium groups were matched for age and sex only. There were significant differences in estimated IQ, cognitive scores, and CSF Aβ42, t-tau, p-tau, and p-tau/Aβ42 between control and delirium groups [27].

### 3.2. Analysis of Metabolites

***Changes related to surgery:*** A total of 121 metabolites out of 138 significantly differed between pre-op and post-op samples (*p* < 0.05 and q < 0.05; Supplementary Table S2). Of the 121 metabolites, just 4 metabolites were increased preoperatively to postoperatively (PC aa C24:0: 16.77%, putrescine: 16.68%, spermine: 8.14%, glucose (H1): 8.14%), while all other metabolites were decreased. The five metabolites that changed by the greatest magnitude perioperatively were: SM(OH) C22:1, Met-SO, Tryptophan (Trp), SM(OH)C22:2, and lysoPCaC18:0 (−37.48% for SM(OH) C22:1, −36.13% for Met-SO, −35.82% for Trp, −33.08% for SM (OH) C22:2, −25.95% for lyso PCaC18:0) (Supplementary Figure S1).

**Figure 1:**
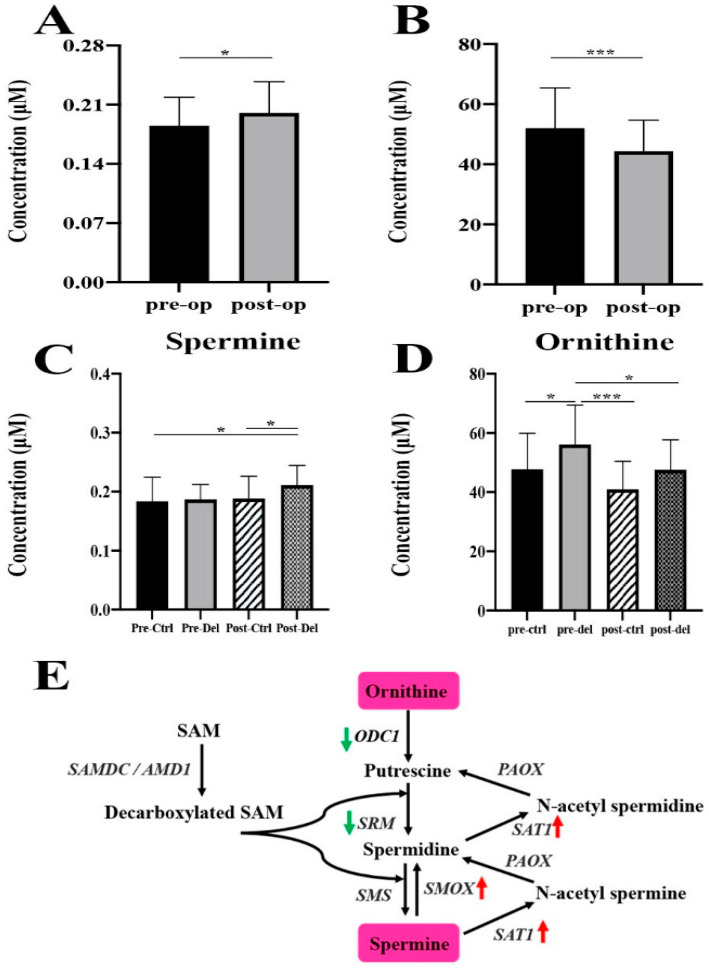
**Plasma levels of spermine and ornithine differ between control and delirium.** Spermine (**A**) and ornithine (**B**) concentrations (μM) in pre- and post-op samples. Spermine (**C**) and ornithine (**D**) concentrations (μM) in pre- and post-op samples with control and delirium. (**E**) shows the relevant metabolic pathway where ornithine is converted into spermine by the action of ornithine decarboxylase (ODC1). * p < 0.05, *** p < 0.001 for both pre- and post-op and pre- and post-op with control and delirium for each group. Pre-op: preoperative; Post-op: postoperative; Pre-ctrl: preoperative control; Pre-del: preoperative delirium; Post-ctrl: postoperative control; Post-del: postoperative delirium.

***Changes related to delirium and surgery:*** A total of 98 of 138 metabolites significantly differed when the data were divided into 4 groups (pre-control, pre-delirium, post-control, and post-delirium (*p* < 0.05, q < 0.05; Table S3)). However, there were only two metabolites that differed between their corresponding control and delirium groups (Figure 1). The first of these was spermine, which was the only metabolite to differ between post-control and post-delirium (increased by 12.0%). The second was ornithine, which was the only metabolite that differed between pre-control and pre-delirium (increased by 17.6%).

**Figure 2:**
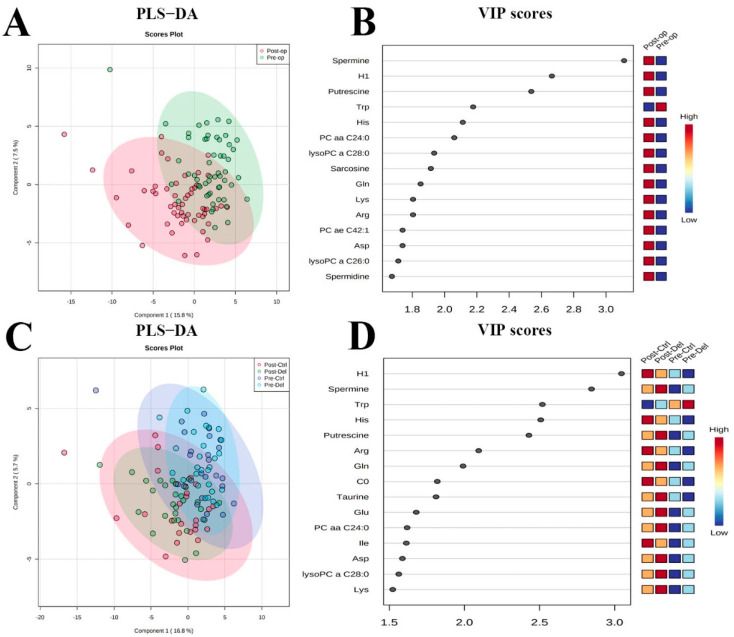
**Multivariate modeling of metabolites by phase of surgery and delirium status.** (**A**) Partial least squares discriminant analysis (PLS-DA) of preoperative and postoperative samples and (**B**) the resulting variable importance in projection (VIP) plot showing the 15 most influential metabolites responsible. (**C**) PLS-DA of control and delirium cases preoperatively and postoperatively and (**D**) resulting VIP showing the 15 most influential metabolites responsible. Pre-op: preoperative; Post-op: postoperative; Pre-ctrl: preoperative control; Pre-del: preoperative delirium; Post-ctrl: postoperative control; Post-del: postoperative delirium.

***Multivariate modeling:*** Multivariate modeling (PLS-DA) clearly differentiated pre-op and post-op plasma samples (Figure 2A; R2 = 0.80, Q2 = 0.38). The 15 most influential metabolites in the VIP plot consisted of 6 amino acids, 4 glycerophospholipids, 4 biogenic amines, and glucose (H1). The top five ranked metabolites were spermine, glucose (H1), putrescine, Trp, and Histidine (His) (Figure 2B). Further modeling (Figure 2C) showed that the phase of surgery differentiated samples to a much greater extent than delirium status (R2 = 0.64; Q2 = 0.25). Here, the 15 most influential metabolites in the VIP plot were 8 amino acids, 3 biogenic amines, 2 glycerophospholipids, 1 acylcarnitine, and glucose (H1). The top five most ranked metabolites were: glucose (H1), spermine, Trp, His, and putrescine (Figure 2D).

Seven metabolites were commonly influential in both models (Trp, H1, putrescine, Gln, Lys, PC aaC24:0, and spermine), and their characteristics are shown in Supplementary Table S2 (mean concentration (μM), standard deviation (SD), *p*-values, q-values, fold change, and % change). 

### 3.3. Predictor Variables for Delirium

To investigate potential predictor variables for delirium, binary logistic regression analysis was undertaken. Table 1. outlines the results of unadjusted and adjusted logistic regression modeling. Perioperative % change in spermine and ornithine were considered for the analysis. CSF Aβ42 shows a significant difference for p-value in both unadjusted and adjusted outcome (unadjusted results: odds ratio for delirium, 0.995; 95% confidence interval (CI), 0.992–0.999; *p*-value, 0.005; adjusted results: odds ratio for delirium, 0.994; 95% (CI), 0.989–0.999; *p*-value, 0.027). Estimated IQ and MMSE show a significant difference for *p*-value in unadjusted results (estimated IQ: odd ratio for delirium, 0.934; 95% (CI), 0.872–0.999; *p*-value, 0.047; MMSE: odd ratio for delirium, 0.711; 95% (CI), 0.528–0.958; *p*-value, 0.025). Neither spermine nor ornithine reached statistical significance. Although, the adjusted result for spermine was just outside the *p*-value threshold (odd ratio for delirium, 1.036; 95% (CI), 0.997–1.076; *p*-value, 0.071).

### 3.4. Associations between Perioperative Variables and Metabolites

Univariate analyses of perioperative variables between delirium and control groups are displayed in Table S4. Excluding antibiotics, there is statistical significance between control and delirium in premedications, anesthesia, painkillers, and others (Supplementary Table S4). Based on those results, the correlation between perioperative variables and % change in all metabolites has been conducted. Perioperative administration of gentamicin is significantly correlated with % change in Glu (q-value: 0.028). Peripheral nerve block/plexus blockade correlated significantly with the perioperative change in 71/138 metabolites, most of which are lipids (Supplementary Table S5). The association between perioperative variables and % change in metabolites was investigated for spermine and ornithine. No perioperative variables correlated with % change in spermine (Table 2). In the case of ornithine, no perioperative variables correlated apart from ‘peripheral nerve block/plexus blockade (yes/no)’ with a q-value of 0.04.

## 4. Discussion

In this investigation, plasma from a nested case–control cohort of postoperative delirium underwent broad metabolite profiling and showed two metabolites, spermine and ornithine, were significantly elevated in delirium cases. The physiological context for the observations was either hip or knee arthroplasty, and the surgical procedures had an overriding impact on the plasma metabolome. The occurrence of delirium influenced the overall metabolome to a minor extent, and furthermore, those changes linked to delirium appear to be highly specific. In blood samples taken preoperatively, ornithine was almost 17.6% higher in persons prone to delirium. Furthermore, a metabolically related metabolite, spermine, was almost 12.0% higher postoperatively in cases of delirium.

The fact that ornithine differed preoperatively, before the ensuing metabolic turbulence of surgery, indicates a possibility that this metabolite is a prognostic biomarker and/or risk factor for delirium. Although logistic regression modeling did not determine ornithine to confer additional risk in this nested cohort, sampling of larger cohorts is needed to definitively determine this risk. Ornithine overall was lowered by the surgical insult, but then this was also the case for the vast majority (138 in total) of measured metabolites. This postsurgical lowering was only significant in delirium cases and not in controls, which is intriguing since ornithine is a precursor for polyamine formation, which includes spermine (but also spermidine and putrescine). Postoperative spermine was also not determined to be a significant risk factor here, but the odds ratio had a p-value of 0.07, indicating that with increased statistical power it possibly could be. The delirium-specific rise in spermine following surgery raises the question of whether the activity of the rate-limiting enzyme, ornithine decarboxylase (ODC) is augmented in individuals prone to delirium. The translation and activity of ODC are regulated by various factors, which include changes in polyamine concentrations [28]. ODC converts ornithine to putrescine, which is then converted to spermidine, and then spermine [28]. The perioperative change in ornithine correlated with only one perioperative factor (i.e., whether nerve blockade was administered or not), and it is possible that this influenced ornithine concentrations postoperatively. Nonetheless, the elevation of ornithine pre-existed before surgery, which is an interesting observation that warrants follow-up in other delirium cohorts. Pharmacological inhibition of ODC activity would be possible if a scientific consensus supported this.

There is a neurological context for the dysregulation of polyamines. Most pertinently, we have recently demonstrated using preoperatively collected cerebrospinal fluid (CSF) from this same cohort that spermidine, putrescine, and glutamine were significantly higher in delirium patients compared with controls [15]. It should be noted that polyamines act physiologically to improve and preserve memory function during aging with actions on neuronal function and axonal integrity [29,30,31,32]. Within the context of neurodegenerative diseases such as Alzheimer’s disease (AD), Parkinson’s disease (PD), and dementia with Lewy bodies (DLB), the dysregulation of polyamines has been evidenced [33,34,35,36,37], but their role remains unclear. The elevation of polyamine pathway metabolites has previously been reported in brain tissue, plasma, and CSF from AD patients [33,34,35,36]. Plasma levels of spermine and the spermine/spermidine ratio were significantly reduced in PD [37]. In neocortical postmortem brain tissue, the levels of putrescine were 55% lower in cases of DLB compared with age-matched controls [38]. There is evidence that an imbalance in polyamines may exacerbate the progression of neurodegenerative disease by contributing to tau neuropathology and cognitive impairment [39].

The aggregation of Aβ and phosphorylated tau protein are considered hallmarks of AD. The present investigation did not find evidence of CSF t-tau or p-tau associated with delirium; however, the data from the entire cohort did find a relationship [27], so the lack of confirmation here may be the result of reduced statistical power. However, we demonstrated that lower CSF concentrations of Aβ42 represent a significantly increased risk of delirium (adjusted odds ratio = 0.994; *p* = 0.027). Aβ is a pathological feature of AD, and CSF Aβ is used for specific diagnosis of AD. Lower CSF Aβ42 normally signifies greater aggregation of Aβ in amyloid plaques in the brain [40]. This study supports earlier work showing an independent association between lower CSF Aβ42 and incidence of postoperative delirium [27]. Previous research has attributed upregulated polyamine metabolism to Aβ aggregation [41,42]. Furthermore, putrescine, spermidine, and spermine have all been shown to interact with Aβ peptides, influencing their aggregation towards the formation of amyloid plaques [43]. It is noteworthy that Aβ peptides increase the activity of ODC, which increases polyamine synthesis [42,44].

The delirium and control groups in this case–control study were matched only for age and sex and differed in a number of other significant ways. Supplementary Table S1, for example, shows the differences in cognitive tests between the groups that were significant in this sub-study, and there are further statistically significant differences, as with CSF tau, when the whole cohort is considered [26,27]. There are statistically significant differences in perioperative factors between the delirium and no delirium groups, as shown in Supplementary Table S4. These may be due to chance, given the multiple comparisons, may reflect changes in the anesthetic approach to participants who were judged preoperatively to be more cognitively vulnerable, and/or may be spurious and influenced by missing data, which though small in number may be significant given the small numbers of events reported in Supplementary Table S4. These perioperative variables did not, with the minor exception of peripheral nerve blockade and gentamicin administration, correlate significantly with perioperative changes in circulating metabolites, suggesting that perioperative metabolite changes can be considered independent from these perioperative factors.

Despite the relatively small sample size of this nested case–control study, which will have limited the statistical power, there are a number of key strengths to this investigation. Firstly, the collection of preoperative and postoperative blood samples is relatively unique; secondly, the detailed clinical characterization and measurement of CSF biomarkers; thirdly, the collection of perioperative information helps exclude potential confounding from the surgical and anesthetic procedures; and finally, the acquisition of annotated and quantitative metabolomic data provides a relatively broad understanding of the impact on metabolism. There are also some minor limitations. The nested case–control used available blood samples from participants matched only for age and sex who did and did not develop delirium following elective arthroplasty. A very small number of participants lacked perioperative variable data (Supplementary Table S4). The scarcity of other comparable delirium cohorts has meant that it has not been possible to externally validate the results. Furthermore, we acknowledge that the logistics of sample collection and storage are potential limitations of this study. Although samples were kept on ice during that period and were treated in a highly consistent manner, some alteration or degradation of metabolites in the samples cannot be ruled out. Future studies should either record the precise duration of the delay or ensure that this time is fixed.

In summary, metabolomic approaches are providing new insights underlying delirium occurrence, allowing new hypotheses to be formed. Once again, the findings here indicate that polyamine metabolism either contributes to the development of delirium or is a part of underlying neurodegeneration or vulnerability. Clearly, a comprehensive and focused examination of the entire arginine–ornithine biochemical pathway is now warranted. This should include the measurement of metabolites from various tissue compartments involved in the urea cycle, polyamine biosynthesis (including their acetylated forms), as well as interconnecting metabolites such as agmatine and glutamine. The most logical way forward should be to establish a bespoke targeted quantitative LC-MS/MS methodology for measuring all the metabolites in the wider biochemical pathway. Then, this methodology could be applied to samples acquired from a range of different postoperative delirium cohorts.

## Figures and Tables

**Table 1:** Binary logistic regression analysis of predicting variables for delirium.

Variables	Unadjusted			Adjusted		
	Odds Ratio	95% CI	*p*-Value	Odds Ratio	95% CI	*p*-Value
Spermine (% change)	1.019	0.995–1.043	0.126	1.036	0.997–1.076	0.071
Ornithine (% change)	1.023	0.983–1.064	0.263	1.037	0.964–1.116	0.327
Age	1.012	0.916–1.118	0.820	0.969	0.842–1.114	0.658
Sex (F/M)	1.000	0.336–2.976	1.000	0.709	0.132–3.815	0.689
Surgery Type (hip vs. knee)	2.879	0.948–8.744	0.062	1.741	0.295–10.281	0.540
CCI	1.618	0.776–3.372	0.199	1.431	0.425–4.816	0.563
Estimated IQ	0.934	0.872–0.999	0.047 *	0.946	0.852–1.051	0.302
MMSE	0.711	0.528–0.958	0.025 *	0.871	0.578–1.311	0.507
Aβ42	0.995	0.992–0.999	0.005 **	0.994	0.989–0.999	0.027 *

Significant *p*-values are shown in bold. * *p* < 0.05, ** *p* < 0.01 variables vs. delirium. CI: confidence interval; CCI: Charlson Comorbidity Index; MMSE: Mini-Mental State Examination; Aβ42: amyloid beta42.

**Table 2:** The association between perioperative variables and % change in spermine and ornithine.

	Spermine	Ornithine
	q-Value	q-Value
**Methods and infusions**		
Length of surgical time (min) (*n* = 23)	1.000	1.000
Intraoperative blood loss (mL) (*n* = 46)	0.193	0.237
Total intraoperative fluid volume (mL) (*n* = 46)	0.922	0.426
Hartmann’s volume (mL) (*n* = 41)	0.930	0.930
Tetraspan/gelofusine volume (mL) (*n* = 39)	0.477	0.736
**Premedications**		
Multi-premedication (yes/no) (*n* = 48)	0.985	0.985
PreMed-Ranitidine (yes/no) (*n* = 48)	0.878	0.878
PreMed-PPI (yes/no) (*n* = 48)	0.962	0.878
PreMed-Benzodiazepine (yes/no) (*n* = 48)	0.975	0.970
**Anesthesia**		
General Anesthesia (yes/no) (*n* = 54)	0.385	0.242
Perioperative iv midazolam (yes/no) (*n* = 47)	0.993	0.993
Intraoperative sedation (propofol) (yes/no) (*n* = 47)	0.907	0.907
Ketamine (yes/no) (*n* = 48)	0.138	0.887
**Painkillers**		
Intravenous IV opioid (yes/no) (*n* = 47)	0.986	0.690
Peripheral nerve block/plexus blockade (yes/no) (*n* = 47)	0.571	0.040 *
Analgesics in recovery (yes/no) (*n* = 46)	0.802	0.802
Intrathecal diamorphine dose (μg) (*n* = 48)	0.913	0.976
**Antibiotics**		
Fluclox (yes/no) (*n* = 46)	1.000	1.000
Cefuroxime (yes/no) (*n* = 46)	0.998	0.998
Teicoplanin (yes/no) (*n* = 46)	0.967	0.967
Gentamicin (yes/no) (*n* = 46)	0.994	0.994
**Others**		
Intraoperative tachycardia (yes/no) (*n* = 45)	0.581	0.134
Intraoperative hypotension (yes/no) (*n* = 45)	0.995	0.995
Ephedrine (yes/no) (*n* = 48)	0.981	0.947
Dexamethasone (yes/no) (*n* = 48)	0.999	0.999
Chlorphenamine (yes/no) (*n* = 48)	0.946	0.946
Magnesium Sulfate (yes/no) (*n* = 48)	0.946	0.946
Ondansetron (yes/no) (*n* = 48)	0.993	0.993
Prochlorperazine (yes/no) (*n* = 48)	0.996	0.996

q-values are from Benjamini–Hochberg, and significant q-values are in bold and present * q < 0.05, perioperative clinical factors vs. % change in metabolites. *n* = numbers of participants out of 54 with data available. PPI; Proton-pump inhibitor. Multi-premedication includes ranitidine, PPI, and benzodiazepine.

## Data Availability

Data is contained within the article or supplementary material.

## Supplementary Materials

The following supporting information can be downloaded at: https://www.mdpi.com/article/10.3390/metabo12070616/s1, Table S1: Baseline characteristics of control and delirium groups; Table S2: Univariate analysis of all the metabolites between pre and post-op; Table S3: Univariate analysis of all the metabolites between pre and post-op with control and delirium; Table S4: Univariate analysis of perioperative variables between control and delirium; Table S5: The association between perioperative variables and % change in all metabolites; Figure S1: The % change rate of 121 metabolites between pre- and post-op.
