# Supplementary material for: The Influence of Orthopedic Surgery on Circulating Metabolite Levels, and their Associations with the Incidence of Postoperative Delirium
Source: Metabolites. 2022 Jul 1;12(7):616. doi: 10.3390/metabo12070616 (PMC9319890; doi:10.3390/metabo12070616)
Supplement: Supplementary file 1 [file metabolites-12-00616-s001.zip › metabolites-1726004-supplementary.pdf]

Supplementary Table S1. Baseline characteristics of control and delirium groups.

	Control (*n*=26)	Delirium (*n*=28)	Statistical test	*p*-Value
Age, mean (SD)	75.8 (5.2)	76.2 (5.7)	T= -0.224	0.82
Sex, female (%)	14/26	15/28	X2= 0	1.00
Type of surgery (hip vs knee), hip (%)	15 out of 26	9 out of 28	X2=2.604	0.11
Years in Education, mean (SD)	11.4 (2.3)	11.3 (1.8)	MWU= 363	0.985
BADL Score (Range:0-9), median (IQR)	3 (1-3)	3 (1-3)	MWU=356	0.884
Vertical Visual Analogue Pain Scale pain at rest, mean (SD)	26.5 (22.7)	19.7 (24.9)	MWU=260.5	0.107
Vertical Visual Analogue Pain Scale pain on movement, mean (SD)	75.7 (14.7)	65.0 (24.0)	T= 1.969	0.055
Estimated IQ, mean (SD)	111.7 (7.5)	106.8 (9.3)	T=2.093	**0.041***
CLOX 1 score, (Range:6-15), median (IQR)	13 (11-14)	12 (11-13)	MWU=303.5	0.286
Letter fluency mean, mean (SD)	11.5 (3.9)	10.4 (4.2)	T= 1.077	0.286
Category fluency mean, mean (SD)	16.3 (3.8)	15.2 (3.7)	T= 1.106	0.274
Stroop colour-word score, mean (SD)	22.3 (6.1)	20.1 (6.8)	T= 1.247	0.218
Time taken to complete Colour Trails 2 (seconds), mean (SD)	146.4 (50.2)	205.2 (115.1)	MWU=235.5	**0.041***
New York university paragraph recall test (immediate recall score), (Range:1-9), median (IQR)	5.44 (3.83-6.89)	4.00 (3.00-5.27)	MWU=237.5	**0.026***
New York university paragraph recall test (delayed recall score), (Range:0-12), median (IQR),	5 (3-8)	4 (2-5.75)	MWU=251.5	0.050
American Society Anesthesiologists (ASA) physical status (I/II/III)	0/22/3	1/21/6	MWU=328	0.564
Charlson Comorbidity Index (CCI) 0/1/2/3	15/8/3/0	11/11/4/1	MWU=286.5	0.209
Mini Mental State Examination Score (MMSE), mean (SD)	27.7 (1.9)	26.0 (2.8)	MWU= 203	**0.012***
Aβ42 in CSF, mean (SD)	613.88 (190.34)	453.01 (175.77)	T=3.198	**0.002****
t-tau in CSF, mean (SD)	291.32 (181.73)	401.01 (233.02)	MWU=204	**0.009****
p-tau in CSF, mean (SD)	51.55 (21.98)	63.88 (23.45)	MWU=201	**0.008****
p-tau/Aβ42 in CSF, man (SD)	0.10 (0.08)	0.17 (0.09)	MWU=122	**4.60E-05*****

Significant *p*-values are shown in bold. *p < 0.05, **p < 0.01, *** p < 0.001 control vs. delirium. CCI: Charlson Comorbidity Index; BADL: Bristol activities of daily living scale; CLOX: clock drawing test; IQR: Interquartile range; ASA: American Society Anesthesiologists; MMSE: Mini mental state examination; SD: standard deviation; T: Student's t.test; X2: Chi-square test; MWU: Mann-Whitney U test.

Supplementary Table S2. Univariate analysis of all the metabolites between pre and post-op.

No.	Name	Pre-op	Post-op	*p*-Value	q-Value	Fold change	% change
		Mean (SD)	Mean (SD)				
**1**	**C0**	**37.117 (7.851)**	**34.859 (8.284)**	**0.0145***	**0.0175***	-1.06	-6.08
2	C14:1	0.2550 (0.0697)	0.2216 (0.0683)	**0.0033****	**0.0040****	-1.15	-13.11
3	C14:2	0.0425 (0.0213)	0.0389 (0.0128)	0.3882	0.4185	-1.09	-8.50
4	C16	0.1252 (0.0353)	0.0987 (0.0283)	**2.00E-05*****	**2.91E-05*****	-1.27	-21.15
5	C16:1	0.0410 (0.0193)	0.0347 (0.0136)	**0.0133***	**0.0162***	-1.18	-15.45
6	C18	0.0380 (0.0142)	0.0321 (0.0137)	0.9300	0.9437	-1.18	-15.59
7	C18:1	0.1699 (0.0531)	0.1329 (0.0358)	**5.53E-08*****	**1.12E-07*****	-1.28	-21.74
8	C2	8.429 (3.594)	6.817 (2.580)	**0.0001*****	**0.0002*****	-1.24	-19.13
9	C3	0.2251 (0.0899)	0.1962 (0.0795)	**0.0175***	**0.0206***	-1.15	-12.86
10	C5	0.1139 (0.0495)	0.1124 (0.0460)	0.8700	0.8894	-1.01	-1.35
11	C5:1-DC	0.0205 (0.0116)	0.0219 (0.0201)	0.8001	0.8302	1.07	6.76

12	C7-DC	0.0263 (0.0118)	0.0224 (0.0113)	**0.0182***	**0.0211***	-1.17	-14.77
13	Ala	339.852 (82.072)	309.259 (87.565)	**9.03E-11*****	**4.02E-10*****	-1.10	-9.00
14	Arg	82.444 (16.322)	79.122 (17.331)	**3.49E-10*****	**1.22E-09*****	-1.04	-4.03
15	Asn	37.856 (6.583)	33.211 (6.366)	**6.36E-10*****	**1.94E-09*****	-1.14	-12.27
16	Asp	3.950 (2.218)	4.314 (2.111)	0.0679	0.0762	1.09	9.24
17	Cit	33.932 (7.907)	31.426 (7.261)	**3.22E-09*****	**8.08E-09*****	-1.08	-7.38
18	Gln	727.778 (80.901)	689.185 (109.631)	**4.20E-05*****	**5.91E-05*****	-1.06	-5.30
19	Glu	41.863 (18.310)	47.680 (21.796)	**0.0444***	0.0502	1.14	13.89
20	Gly	217.630 (81.782)	186.906 (67.247)	**4.21E-07*****	**7.17E-07*****	-1.16	-14.12
21	His	71.702 (8.210)	69.993 (9.867)	**0.0002*****	**0.0003*****	-1.02	-2.38
22	Ile	68.067 (13.860)	65.019 (15.196)	**1.36E-07*****	**2.54E-07*****	-1.05	-4.48
23	Leu	135.994 (34.073)	120.206 (27.714)	**4.40E-05*****	**6.13E-05*****	-1.13	-11.61
24	Lys	167.074 (25.474)	159.135 (28.068)	**3.34E-08*****	**7.09E-08*****	-1.05	-4.75
25	Met	20.970 (3.360)	19.409 (3.757)	**0.0016****	**0.0020****	-1.08	-7.44
26	Orn	52.052 (13.385)	44.398 (10.276)	**2.05E-08*****	**4.42E-08*****	-1.17	-14.70
27	Phe	59.019 (9.523)	52.106 (10.498)	**1.72E-09*****	**4.86E-09*****	-1.13	-11.71
28	Pro	188.019 (35.651)	169.074 (32.092)	**2.17E-11*****	**1.11E-10*****	-1.11	-10.08
29	Ser	86.093 (18.829)	78.474 (17.597)	**3.08E-07*****	**5.31E-07*****	-1.10	-8.85
30	Thr	92.952 (22.316)	83.239 (22.922)	**8.81E-07*****	**1.46E-06*****	-1.12	-10.45
31	Trp	47.565 (10.172)	30.528 (7.719)	**3.00E-06*****	**4.65E-06*****	-1.56	-35.82
32	Tyr	55.280 (13.346)	48.341 (11.136)	**2.44E-07*****	**4.32E-07*****	-1.14	-12.55
33	Val	195.870 (34.450)	181.315 (36.797)	**1.09E-12*****	**6.84E-12*****	-1.08	-7.43
34	ADMA	0.4156 (0.2037)	0.3999 (0.1437)	0.9760	0.9831	-1.04	-3.79
35	Creatinine	66.485 (26.115)	58.806 (18.885)	**0.0137***	**0.0166***	-1.13	-11.55
36	Kynurenine	2.7307 (0.6931)	2.2793 (0.5139)	**1.05E-07*****	**2.01E-07*****	-1.20	-16.53
37	Met-SO	0.9610 (0.3986)	0.6138 (0.3967)	**6.00E-05*****	**8.20E-05*****	-1.57	-36.13
38	Putrescine	0.1098 (0.0411)	0.1281 (0.0481)	**0.0051****	**0.0063****	1.17	16.68
39	Sarcosine	0.9909 (0.3924)	1.0252 (0.3361)	0.1504	0.1635	1.03	3.46
40	Serotonin	0.0959 (0.0920)	0.0980 (0.1062)	0.9894	0.9894	1.02	2.26
41	Spermidine	0.1626 (0.0917)	0.1551 (0.0365)	0.1201	0.1316	-1.05	-4.61
42	Spermine	0.1852 (0.0336)	0.2002 (0.0370)	**0.0177***	**0.0207***	1.08	8.14
43	t4-OH-Pro	9.7676 (3.8200)	8.0417 (2.5102)	**2.87E-07*****	**5.01E-07*****	-1.21	-17.67
44	Taurine	55.731 (12.820)	52.226 (13.928)	**0.0300***	**0.0342***	-1.07	-6.29
45	SDMA	0.6236 (0.4002)	0.6133 (0.2387)	0.6921	0.7346	-1.02	-1.64
46	lysoPC a C16:0	70.343 (17.236)	53.454 (14.489)	**1.63E-12*****	**9.78E-12*****	-1.32	-24.01
47	lysoPC a C16:1	2.4867 (0.6631)	1.9732 (0.5907)	**5.80E-13*****	**3.81E-12*****	-1.26	-20.65
48	lysoPC a C17:0	1.4574 (0.4787)	1.1094 (0.3656)	**1.03E-11*****	**5.47E-11*****	-1.31	-23.88
49	lysoPC a C18:0	19.126 (5.709)	14.162 (4.590)	**2.27E-10*****	**8.95E-10*****	-1.35	-25.95
50	lysoPC a C18:1	16.827 (4.167)	13.518 (3.809)	**4.07E-10*****	**1.37E-09*****	-1.24	-19.66
51	lysoPC a C18:2	20.539 (5.726)	15.811 (4.651)	**6.45E-10*****	**1.94E-09*****	-1.30	-23.02
52	lysoPC a C20:3	2.2506 (0.5592)	1.7666 (0.5556)	**1.76E-09*****	**4.86E-09*****	-1.27	-21.50
53	lysoPC a C20:4	5.2989 (1.6855)	4.1463 (1.5812)	**3.40E-09*****	**8.38E-09*****	-1.28	-21.75
54	lysoPC a C24:0	0.2877 (0.1329)	0.2774 (0.1562)	0.8195	0.8440	-1.04	-3.61
55	lysoPC a C26:0	0.3730 (0.2477)	0.4269 (0.2575)	0.0719	0.0801	1.14	14.44
56	lysoPC a C26:1	0.3161 (0.1700)	0.3320 (0.2115)	0.4590	0.4910	1.05	5.02
57	lysoPC a C28:0	0.4259 (0.1942)	0.4496 (0.1861)	0.0923	0.1019	1.06	5.57
58	lysoPC a C28:1	0.6581 (0.2870)	0.6325 (0.2691)	0.7112	0.7492	-1.04	-3.89
59	PC aa C24:0	0.1491 (0.0929)	0.1741 (0.0879)	**0.0169***	**0.0201***	1.17	16.77
60	PC aa C28:1	2.0666 (0.5494)	1.6401 (0.4630)	**5.32E-13*****	**3.67E-12*****	-1.26	-20.64
61	PC aa C30:0	3.1761 (0.9207)	2.4087 (0.6805)	**1.35E-16*****	**2.66E-15*****	-1.32	-24.16
62	PC aa C32:0	10.3450 (2.8258)	8.2120 (2.441)	**3.01E-10*****	**1.09E-09*****	-1.26	-20.62
63	PC aa C32:1	16.531 (6.563)	13.421 (5.329)	**3.00E-10*****	**1.09E-09*****	-1.23	-18.82
64	PC aa C32:3	0.2677 (0.0644)	0.2124 (0.0567)	**2.46E-10*****	**9.43E-10*****	-1.26	-20.67

65	PC aa C34:1	220.667 (57.438)	184.644 (50.432)	**1.24E-16*****	**2.66E-15*****	-1.20	-16.32
66	PC aa C34:2	313.444 (82.662)	250.848 (69.393)	**8.57E-17*****	**2.37E-15*****	-1.25	-19.97
67	PC aa C34:3	12.3694 (3.4680)	9.9346 (2.8722)	**6.21E-18*****	**4.28E-16*****	-1.25	-19.68
68	PC aa C34:4	1.0889 (0.3722)	0.8570 (0.3104)	**2.42E-09*****	**6.30E-09*****	-1.27	-21.30
69	PC aa C36:0	2.7461 (0.9444)	2.4156 (0.8343)	**1.56E-07*****	**2.87E-07*****	-1.14	-12.03
70	PC aa C36:1	38.889 (11.114)	32.026 (9.277)	**7.09E-14*****	**6.99E-13*****	-1.21	-17.65
71	PC aa C36:2	173.185 (38.597)	136.102 (33.082)	**1.34E-10*****	**5.78E-10*****	-1.27	-21.41
72	PC aa C36:3	104.572 (24.265)	82.548 (21.077)	**2.29E-11*****	**1.13E-10*****	-1.27	-21.06
73	PC aa C36:4	167.263 (49.356)	133.520 (42.546)	**4.80E-10*****	**1.54E-09*****	-1.25	-20.17
74	PC aa C36:5	22.134 (8.430)	17.750 (6.915)	**5.96E-21*****	**8.22E-19*****	-1.25	-19.81
75	PC aa C36:6	0.6154 (0.1984)	0.4881 (0.1775)	**2.52E-17*****	**8.69E-16*****	-1.26	-20.68
76	PC aa C38:0	2.3172 (0.6347)	1.8250 (0.4890)	**2.35E-09*****	**6.30E-09*****	-1.27	-21.24
77	PC aa C38:3	41.515 (10.820)	33.167 (9.937)	**6.71E-14*****	**6.99E-13*****	-1.25	-20.11
78	PC aa C38:4	88.446 (26.965)	72.270 (24.735)	**2.42E-09*****	**6.30E-09*****	-1.22	-18.29
79	PC aa C38:5	46.031 (12.684)	37.211 (11.497)	**4.92E-15*****	**5.97E-14*****	-1.24	-19.16
80	PC aa C38:6	63.602 (17.157)	51.870 (14.439)	**3.68E-09*****	**8.91E-09*****	-1.23	-18.45
81	PC aa C40:2	0.4787 (0.1232)	0.4154 (0.1185)	**5.80E-05*****	**8.00E-05*****	-1.15	-13.23
82	PC aa C40:3	0.5173 (0.1545)	0.4228 (0.1179)	**5.00E-06*****	**7.58E-06*****	-1.22	-18.27
83	PC aa C40:4	2.6076 (0.6890)	2.2108 (0.6987)	**5.39E-08*****	**1.11E-07*****	-1.18	-15.22
84	PC aa C40:5	8.9807 (2.5309)	7.3330 (2.5341)	**4.89E-09*****	**1.16E-08*****	-1.22	-18.35
85	PC aa C40:6	22.174 (6.204)	17.888 (5.327)	**2.90E-09*****	**7.41E-09*****	-1.24	-19.33
86	PC aa C42:0	0.4497 (0.1404)	0.3732 (0.1264)	**8.52E-08*****	**1.68E-07*****	-1.20	-17.01
87	PC aa C42:1	0.2218 (0.0659)	0.1732 (0.0563)	**8.86E-08*****	**1.72E-07*****	-1.28	-21.90
88	PC aa C42:2	0.1877 (0.0535)	0.1635 (0.0548)	**4.00E-05*****	**5.69E-05*****	-1.15	-12.90
89	PC aa C42:4	0.1863 (0.0465)	0.1591 (0.0609)	**0.0008*****	**0.0011****	-1.17	-14.62
90	PC aa C42:5	0.3007 (0.0864)	0.2488 (0.0770)	**1.73E-09*****	**4.86E-09*****	-1.21	-17.26
91	PC aa C42:6	0.4673 (0.1052)	0.4003 (0.0891)	**9.96E-07*****	**1.59E-06*****	-1.17	-14.34
92	PC ae C30:0	0.3082 (0.1038)	0.2575 (0.0715)	**6.00E-06*****	**8.90E-06*****	-1.20	-16.45
93	PC ae C30:1	0.5427 (0.1625)	0.4431 (0.1246)	**0.0016****	**0.0020****	-1.22	-18.36
94	PC ae C30:2	0.1253 (0.0468)	0.1151 (0.0405)	0.7966	0.8302	-1.09	-8.20
95	PC ae C32:1	2.3387 (0.5896)	1.8496 (0.4862)	**6.96E-12*****	**3.84E-11*****	-1.26	-20.91
96	PC ae C32:2	0.4687 (0.1397)	0.3865 (0.1118)	**3.47E-08*****	**7.26E-08*****	-1.21	-17.54
97	PC ae C34:0	1.2672 (0.3981)	0.9775 (0.2787)	**3.76E-12*****	**2.16E-11*****	-1.30	-22.86
98	PC ae C34:1	7.5220 (2.0333)	6.1437 (1.5489)	**1.14E-08*****	**2.58E-08*****	-1.22	-18.32
99	PC ae C34:2	7.0252 (2.0493)	5.6174 (1.5967)	**4.64E-11*****	**2.21E-10*****	-1.25	-20.04
100	PC ae C34:3	4.6626 (1.5911)	3.5776 (1.2430)	**5.19E-15*****	**5.97E-14*****	-1.30	-23.27
101	PC ae C36:0	0.7013 (0.2164)	0.6013 (0.1668)	**6.00E-06*****	**8.90E-06*****	-1.17	-14.26
102	PC ae C36:1	9.7619 (2.5709)	8.2839 (2.2357)	**3.57E-13*****	**2.59E-12*****	-1.18	-15.14
103	PC ae C36:2	9.7348 (2.5577)	8.1306 (2.1256)	**8.73E-11*****	**4.02E-10*****	-1.20	-16.48
104	PC ae C36:3	4.6719 (1.1745)	3.7483 (1.0496)	**1.46E-08*****	**3.20E-08*****	-1.25	-19.77
105	PC ae C36:4	9.6913 (2.4438)	7.7763 (2.0533)	**7.39E-09*****	**1.70E-08*****	-1.25	-19.76
106	PC ae C36:5	7.9494 (2.4445)	6.2626 (2.0832)	**2.34E-15*****	**3.80E-14*****	-1.27	-21.22
107	PC ae C38:0	0.9891 (0.2971)	0.8423 (0.2556)	**2.21E-13*****	**1.79E-12*****	-1.17	-14.84
108	PC ae C38:1	3.9581 (0.9142)	3.3457 (0.8086)	**9.26E-07*****	**1.52E-06*****	-1.18	-15.47
109	PC ae C38:2	3.6572 (0.8789)	3.0622 (0.7337)	**1.00E-06*****	**1.59E-06*****	-1.19	-16.27
110	PC ae C38:3	4.4846 (1.0367)	3.7607 (0.9841)	**3.55E-10*****	**1.22E-09*****	-1.19	-16.14
111	PC ae C38:4	8.9002 (2.0139)	7.4854 (1.8538)	**1.41E-09*****	**4.14E-09*****	-1.19	-15.90
112	PC ae C38:5	11.1281 (2.8446)	9.0276 (2.2733)	**1.43E-08*****	**3.18E-08*****	-1.23	-18.88
113	PC ae C38:6	4.9241 (1.3637)	3.9028 (1.0751)	**1.21E-13*****	**1.11E-12*****	-1.26	-20.74
114	PC ae C40:1	0.8925 (0.2176)	0.7605 (0.1954)	**5.55E-09*****	**1.30E-08*****	-1.17	-14.80
115	PC ae C40:2	1.7549 (0.5110)	1.4575 (0.3972)	**2.32E-07*****	**4.16E-07*****	-1.20	-16.95
116	PC ae C40:3	1.1284 (0.2785)	0.9608 (0.2545)	**2.20E-05*****	**3.16E-05*****	-1.17	-14.86
117	PC ae C40:4	1.6789 (0.3500)	1.4160 (0.3399)	**2.00E-06*****	**3.14E-06*****	-1.19	-15.66

118	PC ae C40:5	2.7935 (0.6303)	2.3604 (0.5687)	**6.54E-08*****	**1.31E-07*****	-1.18	-15.51
119	PC ae C40:6	3.4591 (0.8866)	2.9048 (0.7314)	**1.45E-10*****	**6.06E-10*****	-1.19	-16.02
120	PC ae C42:1	0.2166 (0.0532)	0.1994 (0.0582)	**0.0237***	**0.0272***	-1.09	-7.92
121	PC ae C42:2	0.3601 (0.0865)	0.3160 (0.0869)	**0.0002*****	**0.0003*****	-1.14	-12.25
122	PC ae C42:3	0.5059 (0.1380)	0.4256 (0.1240)	**1.40E-05*****	**2.06E-05*****	-1.19	-15.87
123	PC ae C42:4	0.6408 (0.1778)	0.5402 (0.1684)	**4.00E-06*****	**6.13E-06*****	-1.19	-15.71
124	PC ae C42:5	1.5347 (0.3955)	1.3116 (0.3392)	**1.27E-07*****	**2.40E-07*****	-1.17	-14.54
125	PC ae C44:3	0.0904 (0.0208)	0.0769 (0.0278)	**0.0017****	**0.0021****	-1.18	-14.99
126	PC ae C44:4	0.3046 (0.0829)	0.2649 (0.0888)	**0.0003*****	**0.0004*****	-1.15	-13.06
127	PC ae C44:5	1.4199 (0.4280)	1.1809 (0.3625)	**9.61E-07*****	**1.56E-06*****	-1.20	-16.83
128	PC ae C44:6	1.0951 (0.3276)	0.8649 (0.2605)	**4.94E-10*****	**1.55E-09*****	-1.27	-21.02
129	SM (OH) C14:1	3.2228 (0.8208)	2.4913 (0.7180)	**1.81E-10*****	**7.35E-10*****	-1.29	-22.70
130	SM (OH) C16:1	1.8613 (0.4953)	1.4075 (0.4255)	**4.68E-10*****	**1.54E-09*****	-1.32	-24.38
131	SM (OH) C22:1	2.9569 (1.6729)	1.8487 (1.1700)	**1.68E-07*****	**3.05E-07*****	-1.60	-37.48
132	SM (OH) C22:2	5.6330 (2.2108)	3.7696 (1.5519)	**4.83E-07*****	**8.13E-07*****	-1.49	-33.08
133	SM C16:0	57.165 (13.475)	43.587 (10.615)	**1.62E-13*****	**1.40E-12*****	-1.31	-23.75
134	SM C16:1	8.3546 (2.1450)	6.2833 (1.5783)	**1.00E-17*****	**4.60E-16*****	-1.33	-24.79
135	SM C18:0	15.101 (3.566)	11.621 (3.073)	**2.48E-15*****	**3.80E-14*****	-1.30	-23.05
136	SM C18:1	6.1657 (1.5907)	4.7296 (1.2667)	**3.01E-15*****	**4.15E-14*****	-1.30	-23.29
137	SM C24:0	10.2911 (2.2144)	7.9402 (1.8936)	**2.99E-13*****	**2.29E-12*****	-1.30	-22.84
138	H1	5446.69 (829.25)	5889.78 (1298.04)	**0.0025****	**0.0032****	1.08	8.14

Significant *p*-values are shown in bold. *p < 0.05, **p < 0.01, *** p < 0.001 pre-op vs post-op. q-values are from Benjamini-Hochberg. Significant q-values are shown in bold. *q < 0.05, **q < 0.01, *** q < 0.001 pre-op vs post-op. Pre-op: pre-operative; Post-op: post-operative; SD: standard deviation; Ala: alanine; Arg: arginine; Asn: asparagine; Asp: aspartate; Cit: citrulline ; Gln: glutamine; Glu: glutamate; Gly: glycine; His: histidine; Ile: isoleucine; Leu: leucine; Lys: lysine; Met: methionine; Orn: ornithine; Phe: phenylalanine; Pro: proline; Ser: serine; Thr: threonine; Trp: tryptophan; Tyr: tyrosine; Val: valine; ADMA: asymmetric dimethylarginine; SDMA:symmetric dimethylarginine; H1: hexose.

Supplementary Table S3. Univariate analysis of all the metabolites between pre and post-op with control and delirium.

No.	Name	Pre-Ctrl	Pre-Del	Post-Ctrl	Post-Del	*p*-Value	q-Value	post-hoc test	% Ctrl	% Del
		Mean (SD)	Mean (SD)	Mean (SD)	Mean (SD)					
**1**	**C0**	**37.93(8.30)**	**36.36(7.49)**	**36.19(8.64)**	**33.63(7.90)**	0.270	0.308	-	-4.59	-7.52
2	C14:1	0.258(0.069)	0.252(0.071)	0.215(0.072)	0.228(0.066)	0.083	0.107	-	-16.79	-9.63
3	C14:2	0.045(0.023)	0.040(0.020)	0.039(0.012)	0.039(0.014)	0.583	0.610	-	-14.10	-2.51
4	C16	0.134(0.035)	0.118(0.035)	0.102(0.026)	0.096(0.031)	**2.51E-04*****	**0.002****	pre-ctrl-post-ctrl; pre-ctrl-post-del	-23.75	-18.38
5	C16:1	0.041(0.016)	0.042(0.022)	0.033(0.012)	0.037(0.015)	0.370	0.408	-	-19.51	-11.81
6	C18	0.040(0.013)	0.037(0.015)	0.036(0.012)	0.029(0.015)	**0.037***	0.051	pre-ctrl-post-del	-10.10	-21.04
7	C18:1	0.172(0.038)	0.168(0.065)	0.129(0.026)	0.137(0.043)	**0.001****	**0.004****	pre-ctrl-post-ctrl; pre-ctrl-post-del; pre-del-post-ctrl	-24.96	-18.66
8	C2	8.810(3.605)	8.076(3.614)	7.520(2.756)	6.165(2.262)	**0.015***	**0.025***	pre-ctrl-post-del	-14.65	-23.67
9	C3	0.208(0.080)	0.241(0.097)	0.191(0.076)	0.201(0.083)	0.232	0.267	-	-7.94	-16.79
10	C5	0.110(0.046)	0.117(0.053)	0.110(0.035)	0.115(0.055)	0.974	0.988	-	-0.18	-2.47
11	C5:1-DC	0.019(0.012)	0.022(0.012)	0.016(0.009)	0.027(0.026)	0.388	0.425	-	-15.46	25.46

12	C7-DC	0.030(0.012)	0.023(0.011)	0.026(0.013)	0.019(0.009)	**0.012***	**0.021***	pre-ctrl-post-del	-11.86	-17.95
13	Ala	346.35(75.14)	333.82(88.97)	298.73(81.47)	319.04(93.28)	0.216	0.250	-	-13.75	-4.43
14	Arg	84.14(15.79)	80.87(16.94)	79.86(16.95)	78.44(17.96)	0.653	0.672	-	-5.08	-3.01
15	Asn	37.65(6.34)	38.05(6.91)	32.95(6.70)	33.46(6.15)	**0.005****	**0.011***	pre-del-post-ctrl; pre-del-post-del	-12.49	-12.06
16	Asp	4.054(2.996)	3.853(1.153)	3.971(1.330)	4.634(2.624)	0.201	0.239	-	-2.06	20.28
17	Cit	33.10(7.11)	34.70(8.64)	31.26(6.82)	31.58(7.77)	0.324	0.364	-	-5.57	-9.00
18	Gln	715.85(67.58)	738.86(91.40)	692.19(108.99)	686.39(112.14)	0.172	0.210	-	-3.30	-7.10
19	Glu	42.04(19.64)	41.70(17.35)	47.13(22.32)	48.19(21.69)	0.548	0.582	-	12.10	15.57
20	Gly	198.88(57.76)	235.04(96.88)	173.23(49.54)	199.61(79.10)	**0.035***	**0.049***	pre-del-post-ctrl	-12.90	-15.07
21	His	72.48(6.81)	70.98(9.39)	71.86(9.75)	68.26(9.83)	0.487	0.525	-	-0.85	-3.83
22	Ile	68.73(14.73)	67.45(13.25)	66.36(13.36)	63.77(16.870)	0.589	0.611	-	-3.44	-5.46
23	Leu	138.123(35.725)	134.018(32.996)	124.665(23.726)	116.064(30.81)	**0.028***	**0.042***	pre-ctrl-post-del	-9.74	-13.40
24	Lys	159.50(23.23)	174.11(25.84)	152.65(25.22)	165.15(29.65)	**0.025***	**0.039***	pre-del-post-ctrl	-4.29	-5.14
25	Met	20.71(2.89)	21.21(3.78)	19.19(3.48)	19.61(4.05)	0.142	0.175	-	-7.35	-7.53
26	Orn	47.69(12.24)	56.10(13.33)	40.98(9.45)	47.57(10.15)	**7.80E-05*****	**0.001****	pre-ctrl-pre-del; pre-del-post-ctrl; pre-del-post-del	-14.06	-15.21
27	Phe	59.79(8.65)	58.30(10.38)	52.89(9.15)	51.38(11.73)	**0.006****	**0.013***	pre-ctrl-post-del	-11.54	-11.88
28	Pro	177.73(32.94)	197.57(35.97)	162.50(25.75)	175.18(36.44)	**0.002****	**0.006****	pre-del-post-ctrl	-8.57	-11.33
29	Ser	83.83(17.05)	88.20(20.43)	76.03(16.15)	80.74(18.85)	0.102	0.129	-	-9.30	-8.45
30	Thr	86.87(19.11)	98.60(23.89)	76.72(18.65)	89.29(25.11)	**0.005****	**0.011***	pre-del-post-ctrl	-11.68	-9.44
31	Trp	46.29(10.15)	48.75(10.24)	30.08(7.90)	30.95(7.67)	**8.76E-15*****	**1.21E-12*****	pre-ctrl-post-ctrl; pre-ctrl-post-del; pre-del-post-ctrl; pre-del-post-del	-35.03	-36.52
32	Tyr	53.97(14.02)	56.50(12.82)	47.94(12.14)	48.71(10.32)	**0.032***	**0.047***	-	-11.16	-13.79
33	Val	194.31(28.09)	197.32(39.93)	183.77(30.40)	179.04(42.32)	0.197	0.238	-	-5.42	-9.27
34	ADMA	0.444(0.229)	0.389(0.178)	0.374(0.148)	0.424(0.137)	0.469	0.510	-	-15.74	8.89
35	Creati-nine	70.08(29.65)	63.15(22.37)	62.71(19.07)	55.18(18.31)	0.207	0.242	-	-10.52	-12.61
36	Kynurenine	2.634(0.687)	2.821(0.699)	2.245(0.463)	2.311(0.564)	**0.003****	**0.008****	pre-del-post-ctrl; pre-del-post-del	-14.78	-18.06
37	Met-SO	0.987(0.394)	0.937(0.408)	0.727(0.436)	0.509(0.330)	**1.74E-04*****	**0.001****	pre-ctrl-post-del; pre-del-post-del	-26.33	-45.72

38	Putrescine	0.107(0.040)	0.113(0.042)	0.120(0.042)	0.135(0.053)	0.131	0.163	-	12.95	19.95
39	Sarcosine	0.924(0.421)	1.053(0.361)	0.885(0.318)	1.155(0.303)	**0.018***	**0.029***	pre-ctrl-post-del	-4.25	9.75
40	Serotonin	0.101(0.104)	0.091(0.081)	0.103(0.113)	0.094(0.102)	0.994	0.994	-	1.49	3.07
41	Spermidine	0.162(0.099)	0.163(0.086)	0.149(0.036)	0.161(0.036)	0.088	0.112	-	-8.39	-1.16
42	Spermine	0.183(0.041)	0.187(0.025)	0.189(0.038)	0.211(0.033)	**0.009****	**0.017***	pre-ctrl-post-del; post-ctrl-post-del	2.78	13.01
43	t4-OH-Pro	9.224(3.056)	10.273(4.410)	7.814(2.070)	8.253(2.882)	**0.039***	0.053	-	-15.28	-19.66
44	Taurine	56.74(14.01)	54.79(11.79)	52.20(12.34)	52.25(15.49)	0.553	0.583	-	-8.00	-4.65
45	SDMA	0.695(0.492)	0.557(0.285)	0.572(0.229)	0.652(0.245)	0.520	0.556	-	-17.65	16.88
46	lysoPC a C16:0	71.70(17.61)	69.09(17.11)	54.15(14.98)	52.80(14.26)	**7.00E-06*****	**1.61E-04*****	pre-ctrl-post-ctrl; pre-ctrl-post-del; pre-del-post-ctrl; pre-del-post-del	-24.47	-23.57
47	lysoPC a C16:1	2.477(0.551)	2.495(0.763)	1.961(0.545)	1.984(0.640)	**0.001****	**0.004****	pre-ctrl-post-ctrl; pre-ctrl-post-del; pre-del-post-ctrl; pre-del-post-del	-20.83	-20.48
48	lysoPC a C17:0	1.427(0.464)	1.485(0.498)	1.069(0.356)	1.147(0.377)	**0.001****	**0.004****	pre-ctrl-post-ctrl; pre-del-post-ctrl; pre-del-post-del	-25.11	-22.79
49	lysoPC a C18:0	19.66(6.48)	18.63(4.95)	14.34(5.41)	13.99(3.76)	**2.20E-05*****	**2.89E-04*****	pre-ctrl-post-ctrl; pre-ctrl-post-del; pre-del-post-ctrl; pre-del-post-del	-27.03	-24.90
50	lysoPC a C18:1	16.49(3.94)	17.14(4.42)	13.50(3.45)	13.54(4.18)	**0.001****	**0.004****	pre-ctrl-post-ctrl; pre-ctrl-post-del; pre-del-post-ctrl; pre-del-post-del	-18.13	-21.03
51	lysoPC a C18:2	20.72(5.55)	20.37(5.99)	16.09(4.71)	15.55(4.67)	**1.66E-04*****	**0.001****	pre-ctrl-post-ctrl; pre-ctrl-post-del; pre-del-post-ctrl; pre-del-post-del	-22.35	-23.65
52	lysoPC a C20:3	2.308(0.600)	2.198(0.524)	1.790(0.595)	1.745(0.527)	**1.96E-04*****	**0.001****	pre-ctrl-post-ctrl; pre-ctrl-post-del; pre-del-post-ctrl;	-22.42	-20.61

								pre-del-post-del		
53	lysoPC a C20:4	5.290(1.850)	5.307(1.552)	4.126(1.660)	4.165(1.535)	**0.002****	**0.007****	pre-del-post-ctrl; pre-del-post-del	-22.01	-21.51
54	lysoPC a C24:0	0.275(0.139)	0.300(0.129)	0.293(0.140)	0.263(0.171)	0.779	0.796	-	6.40	-12.15
55	lysoPC a C26:0	0.282(0.138)	0.458(0.295)	0.421(0.269)	0.433(0.251)	0.067	0.087	-	49.29	-5.48
56	lysoPC a C26:1	0.293(0.097)	0.338(0.217)	0.307(0.162)	0.355(0.250)	0.982	0.989	-	4.99	5.06
57	lysoPC a C28:0	0.382(0.117)	0.466(0.240)	0.432(0.196)	0.466(0.178)	0.329	0.366	-	13.00	-0.09
58	lysoPC a C28:1	0.564(0.164)	0.746(0.347)	0.595(0.230)	0.667(0.301)	0.198	0.238	-	5.63	-10.56
59	PC aa C24:0	0.117(0.056)	0.179(0.111)	0.165(0.082)	0.182(0.094)	**0.032***	**0.047***	pre-ctrl-post-del;	40.63	2.18
60	PC aa C28:1	2.084(0.582)	2.050(0.528)	1.639(0.473)	1.641(0.462)	**0.001****	**0.004****	pre-ctrl-post-ctrl; pre-ctrl-post-del; pre-del-post-ctrl; pre-del-post-del	-21.35	-19.96
61	PC aa C30:0	3.192(0.971)	3.162(0.889)	2.404(0.751)	2.413(0.622)	**8.10E-05*****	**0.001****	pre-ctrl-post-ctrl; pre-ctrl-post-del; pre-del-post-ctrl; pre-del-post-del	-24.67	-23.69
62	PC aa C32:0	10.20(2.89)	10.48(2.81)	8.00(2.48)	8.41(2.03)	**3.77E-04*****	**0.002****	pre-ctrl-post-ctrl; pre-del-post-ctrl; pre-del-post-del	-21.58	-19.75
63	PC aa C32:1	17.22(7.42)	15.89(5.72)	13.81(6.22)	13.06(4.44)	0.052	0.070	-	-19.82	-17.81
64	PC aa C32:3	0.267(0.069)	0.269(0.060)	0.215(0.063)	0.210(0.052)	**1.55E-04*****	**0.001****	pre-ctrl-post-ctrl; pre-ctrl-post-del; pre-del-post-ctrl; pre-del-post-del	-19.39	-21.85
65	PC aa C34:1	224.08(56.52)	217.50(59.13)	186.15(52.63)	183.24(49.23)	**0.010***	**0.018***	pre-ctrl-post-del	-16.92	-15.75
66	PC aa C34:2	332.15(99.46)	296.07(60.02)	262.92(83.49)	239.64(52.23)	**1.15E-04*****	**0.001****	pre-ctrl-post-ctrl; pre-ctrl-post-del; pre-del-post-del	-20.85	-19.06
67	PC aa C34:3	12.91(4.01)	11.87(2.86)	10.27(3.36)	9.62(2.35)	**0.001****	**0.004****	pre-ctrl-post-ctrl; pre-ctrl-post-del; pre-del-post-del	-20.42	-18.94

68	PC aa C34:4	1.133(0.429)	1.048(0.313)	0.887(0.381)	0.830(0.231)	**0.004****	**0.009****	pre-ctrl-post-del	-21.77	-20.82
69	PC aa C36:0	2.780(1.074)	2.714(0.825)	2.372(0.852)	2.456(0.831)	0.283	0.320	-	-14.67	-9.53
70	PC aa C36:1	38.59(9.99)	39.17(12.24)	32.11(9.47)	31.95(9.27)	**0.009****	**0.017***	pre-ctrl-post-del	-16.79	-18.43
71	PC aa C36:2	178.96(46.17)	167.83(29.81)	140.32(40.31)	132.19(24.71)	**6.00E-06*****	**1.61E-04*****	pre-ctrl-post-ctrl; pre-ctrl-post-del; pre-del-post-ctrl; pre-del-post-del	-21.59	-21.23
72	PC aa C36:3	108.20(29.04)	101.21(18.72)	85.00(24.12)	80.27(17.95)	**2.20E-05*****	**2.89E-04*****	pre-ctrl-post-ctrl; pre-ctrl-post-del; pre-del-post-ctrl; pre-del-post-del	-21.44	-20.69
73	PC aa C36:4	171.04(56.17)	163.76(42.82)	135.07(46.61)	132.09(39.21)	**0.003****	**0.008****	pre-ctrl-post-ctrl; pre-del-post-ctrl	-21.03	-19.34
74	PC aa C36:5	22.50(8.43)	21.76(8.57)	18.07(7.37)	17.45(6.58)	**0.037***	0.051	-	-19.69	-19.93
75	PC aa C36:6	0.625(0.209)	0.606(0.192)	0.501(0.203)	0.476(0.153)	**0.008****	**0.015***	pre-ctrl-post-del	-19.90	-21.43
76	PC aa C38:0	2.357(0.653)	2.281(0.627)	1.791(0.467)	1.856(0.515)	**0.001****	**0.004****	pre-ctrl-post-ctrl; pre-ctrl-post-del; pre-del-post-ctrl	-23.99	-18.60
77	PC aa C38:3	43.17(12.70)	39.98(8.68)	34.00(10.95)	32.39(9.02)	**0.001****	**0.004****	pre-ctrl-post-ctrl; pre-ctrl-post-del; pre-del-post-del	-21.22	-18.99
78	PC aa C38:4	90.27(29.84)	86.75(24.43)	73.05(27.61)	71.55(22.23)	**0.010***	**0.018***	-	-19.08	-17.53
79	PC aa C38:5	46.83(13.20)	45.29(12.38)	37.46(12.37)	36.982(10.848)	**0.005****	**0.011***	pre-ctrl-post-ctrl	-20.01	-18.35
80	PC aa C38:6	65.91(18.28)	61.46(16.07)	52.62(14.72)	51.18(14.41)	**0.002*****	**0.007*****	pre-ctrl-post-ctrl; pre-ctrl-post-del	-20.17	-16.73
81	PC aa C40:2	0.465(0.101)	0.492(0.141)	0.405(0.119)	0.425(0.119)	**0.010***	**0.018***	pre-del-post-ctrl	-12.81	-13.60
82	PC aa C40:3	0.491(0.125)	0.542(0.176)	0.393(0.113)	0.450(0.118)	**0.001****	**0.004****	pre-del-post-ctrl	-19.88	-16.92
83	PC aa C40:4	2.652(0.677)	2.566(0.710)	2.225(0.752)	2.198(0.659)	**0.035***	**0.049***	-	-16.11	-14.36
84	PC aa C40:5	9.338(2.706)	8.649(2.357)	7.470(2.814)	7.206(2.288)	**0.004****	**0.010***	pre-ctrl-post-ctrl; pre-ctrl-post-del	-20.01	-16.69
85	PC aa C40:6	22.60(6.69)	21.78(5.81)	17.88(5.52)	17.90(5.25)	**0.002****	**0.006****	pre-ctrl-post-ctrl; pre-ctrl-	-20.91	-17.81

								post-del; pre-del-post-ctrl		
86	PC aa C42:0	0.456(0.146)	0.444(0.137)	0.369(0.109)	0.378(0.143)	**0.022***	**0.035***	-	-19.21	-14.92
87	PC aa C42:1	0.225(0.068)	0.219(0.065)	0.175(0.053)	0.172(0.060)	**0.001****	**0.004****	pre-ctrl-post-ctrl; pre-ctrl-post-del; pre-del-post-del	-22.21	-21.62
88	PC aa C42:2	0.191(0.062)	0.185(0.045)	0.157(0.065)	0.169(0.043)	**0.016***	**0.026***	pre-ctrl-post-ctrl; pre-del-post-ctrl	-17.47	-8.54
89	PC aa C42:4	0.180(0.045)	0.192(0.048)	0.166(0.066)	0.153(0.056)	**0.035***	**0.049***	-	-7.79	-20.58
90	PC aa C42:5	0.280(0.085)	0.320(0.085)	0.247(0.079)	0.250(0.076)	**0.004****	**0.010***	pre-del-post-ctrl; pre-del-post-del	-11.68	-21.79
91	PC aa C42:6	0.455(0.103)	0.479(0.108)	0.401(0.086)	0.400(0.093)	**0.005****	**0.011***	pre-del-post-ctrl; pre-del-post-del	-11.89	-16.49
92	PC ae C30:0	0.298(0.105)	0.317(0.103)	0.259(0.086)	0.256(0.057)	**0.043***	0.058	-	-13.27	-19.22
93	PC ae C30:1	0.522(0.140)	0.562(0.181)	0.430(0.104)	0.455(0.142)	**0.004****	**0.010***	pre-del-post-ctrl; pre-del-post-del	-17.63	-18.98
94	PC ae C30:2	0.110(0.032)	0.139(0.054)	0.113(0.035)	0.117(0.046)	0.055	0.073	-	2.72	-16.21
95	PC ae C32:1	2.333(0.641)	2.344(0.550)	1.844(0.559)	1.855(0.418)	**1.85E-04*****	**0.001****	pre-ctrl-post-ctrl; pre-ctrl-post-del; pre-del-post-ctrl; pre-del-post-del	-20.96	-20.87
96	PC ae C32:2	0.451(0.141)	0.486(0.139)	0.367(0.102)	0.405(0.119)	**0.005****	**0.011***	pre-del-post-ctrl	-18.65	-16.58
97	PC ae C34:0	1.227(0.357)	1.305(0.436)	0.954(0.289)	1.000(0.272)	**0.001****	**0.004****	pre-ctrl-post-ctrl; pre-del-post-ctrl; pre-del-post-del	-22.23	-23.40
98	PC ae C34:1	7.394(1.970)	7.641(2.119)	5.927(1.387)	6.345(1.685)	**0.002****	**0.006****	pre-ctrl-post-ctrl; pre-del-post-ctrl; pre-del-post-del	-19.83	-16.97
99	PC ae C34:2	7.223(2.352)	6.841(1.746)	5.585(1.593)	5.648(1.629)	**0.002****	**0.006****	pre-ctrl-post-ctrl; pre-ctrl-post-del	-22.68	-17.45
100	PC ae C34:3	4.750(1.768)	4.581(1.436)	3.551(1.218)	3.603(1.288)	**0.003****	**0.008****	pre-ctrl-post-del	-25.25	-21.37
101	PC ae C36:0	0.739(0.228)	0.666(0.203)	0.602(0.174)	0.601(0.163)	**0.032***	**0.047***	pre-ctrl-post-del	-18.64	-9.76
102	PC ae C36:1	9.473(2.362)	10.030(2.766)	7.882(1.948)	8.657(2.449)	**0.009****	**0.017***	pre-del-post-ctrl	-16.80	-13.69

103	PC ae C36:2	9.617(2.613)	9.844(2.548)	7.868(2.046)	8.375(2.206)	**0.007****	**0.014***	pre-del-post-ctrl	-18.19	-14.93
104	PC ae C36:3	4.856(1.356)	4.501(0.970)	3.755(1.050)	3.742(1.069)	**3.26E-04*****	**0.002****	pre-ctrl-post-ctrl; pre-ctrl-post-del; pre-del-post-del	-22.66	-16.87
105	PC ae C36:4	10.01(2.76)	9.40(2.12)	7.83(2.23)	7.73(1.91)	**3.26E-04*****	**0.002****	pre-ctrl-post-ctrl; pre-ctrl-post-del; pre-del-post-ctrl; pre-del-post-del	-21.80	-17.74
106	PC ae C36:5	8.064(2.764)	7.844(2.152)	6.201(2.314)	6.320(1.885)	**0.002****	**0.006****	pre-ctrl-post-ctrl; pre-del-post-ctrl	-23.10	-19.42
107	PC ae C38:0	0.986(0.324)	0.992(0.276)	0.840(0.262)	0.845(0.254)	0.065	0.085	-	-14.83	-14.84
108	PC ae C38:1	4.036(1.082)	3.886(0.739)	3.331(0.887)	3.360(0.744)	**0.005****	**0.011***	pre-ctrl-post-ctrl; pre-ctrl-post-del;	-17.48	-13.54
109	PC ae C38:2	3.662(0.991)	3.653(0.780)	3.008(0.722)	3.113(0.754)	**0.005****	**0.011***	pre-del-post-ctrl	-17.85	-14.79
110	PC ae C38:3	4.556(1.079)	4.419(1.011)	3.670(0.878)	3.845(1.083)	**0.004****	**0.010***	pre-ctrl-post-ctrl; pre-del-post-ctrl	-19.44	-12.98
111	PC ae C38:4	8.901(2.105)	8.900(1.965)	7.289(1.784)	7.668(1.931)	**0.003****	**0.008****	pre-ctrl-post-ctrl; pre-del-post-ctrl	-18.11	-13.84
112	PC ae C38:5	11.32(3.18)	10.95(2.54)	8.88(2.24)	9.17(2.33)	**0.001****	**0.004****	pre-ctrl-post-ctrl; pre-del-post-ctrl	-21.63	-16.23
113	PC ae C38:6	5.013(1.391)	4.841(1.358)	3.869(0.995)	3.934(1.162)	**0.001****	**0.004****	pre-ctrl-post-ctrl; pre-ctrl-post-del; pre-del-post-ctrl; pre-del-post-del	-22.83	-18.74
114	PC ae C40:1	0.886(0.240)	0.898(0.199)	0.756(0.204)	0.765(0.190)	**0.016***	**0.026***	-	-14.72	-14.85
115	PC ae C40:2	1.744(0.467)	1.766(0.557)	1.417(0.312)	1.495(0.465)	**0.007****	**0.014***	pre-ctrl-post-ctrl	-18.72	-15.32
116	PC ae C40:3	1.147(0.303)	1.111(0.258)	0.948(0.241)	0.972(0.270)	**0.014***	**0.024***	-	-17.31	-12.49
117	PC ae C40:4	1.714(0.381)	1.646(0.323)	1.390(0.315)	1.440(0.366)	**0.002****	**0.006****	pre-ctrl-post-ctrl; pre-ctrl-post-del; pre-del-post-ctrl	-18.89	-12.53
118	PC ae C40:5	2.832(0.618)	2.758(0.650)	2.307(0.511)	2.410(0.623)	**0.004****	**0.010***	pre-ctrl-post-ctrl; pre-del-post-ctrl	-18.51	-12.64

119	PC ae C40:6	3.479(0.929)	3.441(0.862)	2.842(0.682)	2.964(0.782)	**0.007****	**0.014***	pre-ctrl-post-ctrl; pre-del-post-ctrl	-18.32	-13.87
120	PC ae C42:1	0.211(0.050)	0.222(0.056)	0.195(0.057)	0.204(0.060)	0.207	0.242	-	-7.50	-8.24
121	PC ae C42:2	0.369(0.094)	0.352(0.079)	0.310(0.094)	0.321(0.081)	**0.026***	**0.040***	-	-15.82	-8.77
122	PC ae C42:3	0.523(0.163)	0.490(0.111)	0.427(0.139)	0.425(0.110)	**0.008****	**0.015***	pre-ctrl-post-del	-18.50	-13.25
123	PC ae C42:4	0.684(0.190)	0.601(0.158)	0.557(0.193)	0.525(0.144)	**0.007****	**0.014***	pre-ctrl-post-del	-18.66	-12.59
124	PC ae C42:5	1.567(0.448)	1.505(0.346)	1.308(0.338)	1.315(0.346)	**0.021***	**0.033***	-	-16.56	-12.57
125	PC ae C44:3	0.093(0.019)	0.088(0.022)	0.075(0.026)	0.078(0.030)	**0.016***	**0.026***	pre-ctrl-post-ctrl	-18.92	-11.02
126	PC ae C44:4	0.309(0.102)	0.301(0.061)	0.273(0.085)	0.258(0.093)	**0.034***	**0.049***	pre-del-post-del	-11.66	-14.39
127	PC ae C44:5	1.490(0.472)	1.355(0.380)	1.163(0.346)	1.198(0.382)	**0.011***	**0.019***	pre-ctrl-post-ctrl	-21.95	-11.61
128	PC ae C44:6	1.128(0.349)	1.065(0.309)	0.871(0.235)	0.860(0.286)	**0.002****	**0.006****	pre-ctrl-post-ctrl; pre-ctrl-post-del; pre-del-post-del	-22.80	-19.27
129	SM (OH) C14:1	3.197(0.802)	3.247(0.852)	2.499(0.732)	2.484(0.718)	**1.50E-04*****	**0.001****	pre-ctrl-post-ctrl; pre-ctrl-post-del; pre-del-post-ctrl; pre-del-post-del	-21.82	-23.50
130	SM (OH) C16:1	1.835(0.476)	1.886(0.520)	1.406(0.391)	1.409(0.462)	**3.10E-05*****	**3.57E-04*****	pre-ctrl-post-ctrl; pre-ctrl-post-del; pre-del-post-ctrl; pre-del-post-del	-23.37	-25.29
131	SM (OH) C22:1	3.155(1.906)	2.773(1.435)	2.181(1.298)	1.540(0.960)	**0.001****	**0.004****	pre-ctrl-post-del; pre-del-post-del	-30.88	-44.45
132	SM (OH) C22:2	5.565(2.194)	5.696(2.265)	4.097(1.675)	3.466(1.390)	**2.30E-05*****	**2.89E-04*****	pre-ctrl-post-ctrl; pre-ctrl-post-del; pre-del-post-ctrl; pre-del-post-del	-26.38	-39.16
133	SM C16:0	57.12(15.25)	57.20(11.88)	44.01(11.53)	43.19(9.89)	**2.00E-06*****	**1.38E-04*****	pre-ctrl-post-ctrl; pre-ctrl-post-del; pre-del-post-ctrl; pre-del-post-del	-22.95	-24.49
134	SM C16:1	8.552(2.487)	8.172(1.798)	6.444(1.670)	6.134(1.504)	**9.00E-06*****	**1.73E-04*****	pre-ctrl-post-ctrl; pre-ctrl-	-24.64	-24.94

								post-del; pre-del-post-ctrl; pre-del-post-del		
135	SM C18:0	15.50(3.71)	14.73(3.45)	11.98(2.89)	11.29(3.25)	**6.00E-06*** **	**1.61E-04*** **	pre-ctrl-post-ctrl; pre-ctrl-post-del; pre-del-post-ctrl; pre-del-post-del	-22.72	-23.36
136	SM C18:1	6.410(1.713)	5.939(1.463)	4.906(1.176)	4.566(1.346)	**1.00E-05*** **	**1.73E-04*** **	pre-ctrl-post-ctrl; pre-ctrl-post-del; pre-del-post-ctrl; pre-del-post-del	-23.46	-23.12
137	SM C24:0	10.42(2.53)	10.18(1.92)	8.18(2.01)	7.72(1.79)	**3.00E-06*** **	**1.38E-04*** **	pre-ctrl-post-ctrl; pre-ctrl-post-del; pre-del-post-ctrl; pre-del-post-del	-21.47	-24.15
138	H1	5568.88(686.17)	5333.21(941.35)	5984.62(1183.25)	5801.71(1412.21)	0.107	0.134	-	7.47	8.78

Significant *p*-values are shown in bold. *p < 0.05, **p < 0.01, *** p < 0.001 across 4 groups comparisons (pre-ctrl, pre-del, post-ctrl, and post-del). q-values are from Benjamini-Hochberg. Significant q-values are shown in bold. *q < 0.05, **q < 0.01, *** q < 0.001 across 4 groups comparisons (pre-ctrl, pre-del, post-ctrl, and post-del). Pre-Ctrl: pre-operative control; Pre-Del: pre-operative delirium; Post-Ctrl: post-operative control; Post-Del: post-operative delirium; SD: standard deviation; Ala: alanine; Arg: arginine; Asn: asparagine; Asp: aspartate; Cit: citrulline ; Gln: glutamine; Glu: glutamate; Gly: glycine; His: histidine; Ile: isoleucine; Leu: leucine; Lys: lysine; Met: methionine; Orn: ornithine; Phe: phenylalanine; Pro: proline; Ser: serine; Thr: threonine; Trp: tryptophan; Tyr: tyrosine; Val: valine; ADMA: asymmetric dimethylarginine; SDMA:symmetric dimethylarginine; H1: hexose.

Supplementary Table S4. Univariate analysis of perioperative variables between control and delirium.

Types of drugs	Control	Delirium	*p*-Value
**Premedications**			
Multi-premedication (Yes)	24/26	20/22	**0.043***
PreMed-Ranitidine (Yes)	14/26	9/22	**0.029***
PreMed-PPI (Yes)	8/26	5/22	**0.036***
PreMed-Benzodiazepine (Yes)	13/26	10/22	**0.041***
**Anesthesia**			
General Anesthesia (Yes)	1/26	3/28	0.658
Perioperative iv midazolam (Yes)	16/26	16/21	**0.014***
Intraoperative sedation (propofol) (Yes)	23/26	18/21	**0.023***
Ketamine (Yes)	2/26	2/22	**0.043***
**Painkillers**			
Intravenous IV opioid (Yes)	2/26	6/21	**0.004****
Peripheral nerve block/plexus blockade (Yes)	7/26	9/21	**0.012***
Analgesics (Yes)	3/24	2/22	0.341
**Antibiotics**			
Fluclox (Yes)	18/24	17/22	0.359

Cefuroxime (Yes)	1/24	3/22	0.191
Teicoplanin (Yes)	5/24	2/22	0.198
Gentamicin (Yes)	18/24	17/22	0.359
**Others**			
Intraoperative tachycardia (Yes)	6/25	2/20	**0.025***
Intraoperative hypotension (Yes)	11/25	7/20	**0.043***
Ephedrine (Yes)	7/26	4/22	**0.034***
Dexamethasone (Yes)	12/26	10/22	**0.043***
Chlorphenamine (Yes)	1/26	2/22	**0.033***
Magnesium Sulphate (Yes)	3/26	1/22	**0.030***
Ondansetron (Yes)	22/26	20/22	**0.035***
Prochlorperazine (Yes)	7/26	4/22	**0.034***

Significant *p*-values are shown in bold. *p < 0.05, **p < 0.01 control vs. delirium. Chi-square was conducted for comparison. PPI; Proton-pump inhibitor. Multi-premedication includes ranitidine, PPI, and benzodiazepine.

Supplementary Table S5. The association between perioperative variables and % change in all metabolites.

Metabolites	Infusions			Premedications				Anesthesia				Painkillers		
	**Total intraoperative fluid volume**	**Hartmann's volume**	**Tetraspan/Gelofusion volume**	**Multi-medications (Yes/No)**	**Ranitidine (Yes/No)**	**PPI (Yes/No)**	**Benzodiazepine (Yes/No)**	**General anesthesia (Yes/No)**	**Ketamine (Yes/No)**	**Midazolam (Yes/No)**	**Propofol (Yes/No)**	**Peripheral nerve block/plexus blockade (Yes/No)**	**Opioid (Yes/No)**	**Diamorphine (Yes/No)**
	**q-Value**	**q-Value**	**q-Value**	**q-Value**	**q-Value**	**q-Value**	**q-Value**	**q-Value**	**q-Value**	**q-Value**	**q-Value**	**q-Value**	**q-Value**	**q-Value**
C0	0.802	0.930	0.713	0.996	0.878	0.934	0.970	0.622	0.887	0.993	0.854	0.375	0.690	0.913
C141	0.603	0.977	0.651	0.985	0.878	0.878	0.975	0.778	0.789	0.993	0.907	0.113	0.986	0.977
C142	0.420	0.930	0.725	0.985	0.927	0.962	0.970	0.622	0.518	0.993	0.907	0.217	0.986	0.913
C16	0.375	0.930	0.569	0.996	0.932	0.962	0.970	0.469	0.887	0.997	0.854	**0.009****	0.986	0.913
C161	0.536	0.930	0.745	0.985	0.878	0.878	0.980	0.781	0.958	0.993	0.907	0.171	0.986	0.913
C18	0.807	0.930	0.477	0.985	0.880	0.962	0.970	0.554	0.887	0.993	0.907	**0.024***	0.986	0.913
C181	0.949	0.930	0.477	0.985	0.878	0.878	0.970	0.554	0.892	0.993	0.854	**0.010***	0.986	0.913
C2	0.719	0.930	0.665	0.985	0.878	0.962	0.970	0.554	0.789	0.993	0.962	0.895	0.986	0.913
C3	0.610	0.987	0.569	0.985	0.878	0.962	0.970	0.800	0.909	0.993	0.907	0.833	0.986	0.913
C5	0.528	0.930	0.651	0.985	0.878	0.999	0.975	0.554	0.892	0.993	0.907	0.543	0.986	0.913
C51DC	0.801	0.930	0.665	0.985	0.927	0.962	0.970	0.554	0.789	0.993	0.976	0.814	0.986	0.913
C7DC	0.528	0.930	0.823	0.985	0.878	0.999	0.970	0.991	0.892	0.993	0.854	0.925	0.986	0.913
Ala	0.804	0.930	0.569	0.985	0.878	0.962	0.970	0.362	0.887	0.993	0.907	0.115	0.986	0.913
Arg	0.640	0.930	0.665	0.985	0.878	0.878	0.970	0.850	0.892	0.993	0.854	0.164	0.986	0.913
Asn	0.802	0.930	0.578	0.985	0.878	0.962	0.970	0.885	0.924	0.993	0.854	0.340	0.986	0.913
Asp	0.949	0.930	0.477	0.985	0.878	0.962	0.970	0.315	0.887	0.993	0.962	0.309	0.986	0.913
Cit	0.726	0.930	0.541	0.996	0.878	0.878	0.970	0.882	0.892	0.993	0.976	0.979	0.986	0.994
Gln	0.528	0.800	0.569	0.985	0.878	0.878	0.970	0.554	0.892	0.993	0.907	**0.049***	0.986	0.913
Glu	0.801	0.828	0.569	0.985	0.985	0.962	0.970	0.554	0.887	0.993	0.854	0.053	0.986	0.968
Gly	0.569	0.930	0.736	0.985	0.878	0.937	0.970	0.242	0.944	0.993	0.976	**0.032***	0.986	0.913
His	0.454	0.828	0.726	0.985	0.878	0.878	0.970	0.632	0.892	0.993	0.907	0.166	0.986	0.977
Ile	0.398	0.930	0.846	0.985	0.878	0.962	0.970	0.555	0.892	0.993	0.907	0.424	0.986	0.913
Leu	0.804	0.930	0.569	0.985	0.878	0.962	0.970	0.692	0.892	0.993	0.962	0.166	0.986	0.913
Lys	0.594	0.930	0.941	0.985	0.878	0.937	0.970	0.882	0.789	0.993	0.945	0.232	0.986	0.913
Met	0.610	0.930	0.885	0.996	0.878	0.962	0.970	0.554	0.892	0.993	0.854	0.250	0.986	0.913

Orn	0.426	0.930	0.736	0.985	0.878	0.878	0.970	0.242	0.887	0.993	0.907	**0.040***	0.690	0.976
Phe	0.539	0.930	0.894	0.987	0.878	0.878	0.970	0.778	0.887	0.993	0.907	0.118	0.986	0.913
Pro	0.426	0.930	0.819	0.985	0.878	0.937	0.970	0.555	0.892	0.993	0.907	0.104	0.986	0.913
Ser	0.615	0.930	0.809	0.985	0.878	0.878	0.970	0.554	0.911	0.993	0.854	0.086	0.986	0.913
Thr	0.603	0.930	0.725	0.985	0.878	0.934	0.970	0.554	0.887	0.993	0.907	0.132	0.986	0.913
Trp	0.939	0.930	0.614	0.985	0.878	0.878	0.970	0.555	0.951	0.993	0.976	0.904	0.986	0.913
Tyr	0.610	0.930	0.729	0.985	0.878	0.828	0.970	0.829	0.789	0.993	0.854	0.082	0.986	0.971
Val	0.293	0.828	0.712	0.985	0.878	0.878	0.970	0.764	0.892	0.993	0.854	0.275	0.986	0.913
ADMA	0.804	0.930	0.569	0.985	0.878	0.999	0.975	0.554	0.789	0.993	0.907	0.516	0.986	0.977
Creatine	0.806	0.930	0.728	0.996	0.878	0.962	0.970	0.707	0.937	0.993	0.907	0.372	0.986	0.913
Kynurenine	0.844	0.930	0.925	0.985	0.906	0.962	0.970	0.554	0.789	0.993	0.907	0.262	0.986	0.913
MetSO	0.777	0.930	0.569	0.985	0.878	0.962	0.970	0.746	0.789	0.993	0.907	0.809	0.986	0.986
Putrescine	0.946	0.930	0.477	0.985	0.878	0.999	0.970	0.707	0.892	0.993	0.993	0.609	0.986	0.913
Sarcosine	0.804	0.930	0.569	0.985	0.878	0.999	0.970	0.692	0.892	0.993	0.976	0.347	0.986	0.913
Serotonin	0.426	0.930	0.477	0.985	0.878	0.999	0.970	0.720	0.892	0.993	0.976	0.337	0.986	0.913
Spermidine	0.804	0.951	0.477	0.985	0.963	0.962	0.970	0.554	0.789	0.993	0.907	0.904	0.986	0.994
Spermine	0.922	0.930	0.477	0.985	0.878	0.962	0.975	0.385	0.138	0.993	0.907	0.571	0.986	0.913
t4OHPro	0.426	0.800	0.477	0.985	0.878	0.962	0.970	0.083	0.892	0.993	0.907	**0.010***	0.690	0.913
Taurine	0.967	0.930	0.569	0.996	0.878	0.962	0.970	0.990	0.518	0.993	0.962	0.882	0.986	0.913
SDMA	0.610	0.930	0.477	0.985	0.878	0.962	0.970	0.069	0.924	0.993	0.907	0.159	0.986	0.913
lysoPCaC160	0.426	0.930	0.706	0.985	0.878	0.934	0.970	0.554	0.887	0.993	0.854	**0.043***	0.986	0.913
lysoPCaC161	0.510	0.930	0.665	0.985	0.878	0.962	0.980	0.554	0.892	0.993	0.907	**0.030***	0.986	0.913
lysoPCaC170	0.426	0.930	0.569	0.996	0.878	0.878	0.975	0.468	0.518	0.993	0.907	0.144	0.986	0.913
lysoPCaC180	0.420	0.930	0.665	0.996	0.878	0.878	0.970	0.554	0.887	0.993	0.893	**0.035***	0.986	0.913
lysoPCaC181	0.610	0.930	0.712	0.985	0.878	0.878	0.970	0.685	0.887	0.993	0.907	0.059	0.986	0.913
lysoPCaC182	0.528	0.930	0.665	0.985	0.878	0.878	0.970	0.554	0.892	0.993	0.907	0.056	0.986	0.913
lysoPCaC203	0.399	0.930	0.477	0.985	0.878	0.878	0.970	0.692	0.957	0.993	0.854	**0.040***	0.986	0.913
lysoPCaC204	0.580	0.930	0.569	0.985	0.878	0.878	0.975	0.554	0.892	0.993	0.907	**0.040***	0.986	0.913
lysoPCaC240	0.949	0.930	0.569	0.985	0.878	0.962	0.970	0.554	0.887	0.993	0.854	**0.010***	0.986	0.913
lysoPCaC260	0.909	0.930	0.477	0.985	0.937	0.999	0.980	0.400	0.887	0.993	0.854	0.390	0.986	0.794
lysoPCaC261	0.610	0.930	0.712	0.996	0.878	0.962	0.980	0.069	0.892	0.993	0.907	0.081	0.986	0.635
lysoPCaC280	0.603	0.930	0.477	0.985	0.878	0.999	0.975	0.069	0.892	0.993	0.907	**0.043***	0.986	0.794
lysoPCaC281	0.640	0.930	0.477	0.996	0.927	0.999	0.975	0.237	0.892	0.993	0.976	0.120	0.986	0.414
PCaaC240	0.725	0.930	0.477	0.985	0.878	0.999	0.970	0.069	0.887	0.993	0.907	**0.042***	0.986	0.913

PCaaC281	0.293	0.930	0.836	0.985	0.927	0.999	0.970	0.242	0.887	0.993	0.907	**0.008****	0.986	0.913
PCaaC300	0.420	0.930	0.569	0.996	0.878	0.962	0.970	0.554	0.789	0.993	0.907	**0.024***	0.986	0.913
PCaaC320	0.293	0.930	0.569	0.996	0.878	0.962	0.970	0.554	0.887	0.993	0.907	**0.024***	0.986	0.913
PCaaC321	0.375	0.930	0.569	0.996	0.878	0.962	0.975	0.692	0.789	0.993	0.907	**0.043***	0.986	0.913
PCaaC323	0.375	0.930	0.712	0.985	0.938	0.999	0.980	0.115	0.937	0.993	0.907	**0.024***	0.986	0.913
PCaaC341	0.375	0.930	0.569	0.996	0.878	0.962	0.970	0.692	0.887	0.993	0.907	0.052	0.986	0.913
PCaaC342	0.293	0.930	0.569	0.985	0.878	0.962	0.970	0.622	0.887	0.993	0.893	**0.033***	0.986	0.913
PCaaC343	0.420	0.930	0.569	0.996	0.878	0.962	0.970	0.554	0.887	0.993	0.907	**0.026***	0.986	0.913
PCaaC344	0.528	0.930	0.632	0.985	0.927	0.962	0.980	0.554	0.892	0.993	0.893	**0.030***	0.986	0.913
PCaaC360	0.949	0.930	0.569	0.985	0.937	0.999	0.970	0.727	0.951	0.993	0.907	0.375	0.986	0.913
PCaaC361	0.497	0.930	0.712	0.996	0.878	0.962	0.970	0.554	0.892	0.993	0.907	**0.033***	0.986	0.913
PCaaC362	0.528	0.930	0.665	0.985	0.904	0.977	0.970	0.554	0.892	0.993	0.907	**0.040***	0.986	0.913
PCaaC363	0.439	0.930	0.665	0.985	0.927	0.962	0.970	0.554	0.892	0.993	0.854	**0.024***	0.986	0.913
PCaaC364	0.426	0.930	0.665	0.985	0.910	0.977	0.970	0.692	0.892	0.993	0.893	**0.043***	0.986	0.913
PCaaC365	0.610	0.930	0.758	0.985	0.878	0.999	0.975	0.554	0.937	0.993	0.893	0.054	0.986	0.913
PCaaC366	0.426	0.930	0.665	0.985	0.878	0.962	0.975	0.602	0.892	0.993	0.854	**0.010***	0.986	0.913
PCaaC380	0.510	0.930	0.569	0.985	0.878	0.962	0.975	0.555	0.892	0.993	0.854	**0.046***	0.986	0.913
PCaaC383	0.539	0.930	0.765	0.985	0.878	0.966	0.970	0.554	0.892	0.993	0.854	**0.022***	0.986	0.913
PCaaC384	0.539	0.930	0.765	0.996	0.878	0.962	0.975	0.555	0.892	0.993	0.893	**0.047***	0.986	0.913
PCaaC385	0.569	0.930	0.725	0.996	0.878	0.962	0.970	0.554	0.892	0.993	0.907	0.052	0.986	0.913
PCaaC386	0.564	0.930	0.736	0.985	0.878	0.962	0.980	0.554	0.892	0.993	0.893	**0.036***	0.986	0.913
PCaaC402	0.784	0.930	0.477	0.985	0.878	0.878	0.970	0.555	0.946	0.993	0.854	0.142	0.986	0.913
PCaaC403	0.439	0.930	0.776	0.985	0.878	0.962	0.970	0.340	0.892	0.993	0.854	**0.005****	0.986	0.913
PCaaC404	0.777	0.930	0.569	0.996	0.878	0.878	0.970	0.622	0.892	0.993	0.907	**0.024***	0.986	0.977
PCaaC405	0.528	0.930	0.569	0.996	0.878	0.999	0.975	0.554	0.957	0.993	0.854	0.135	0.986	0.913
PCaaC406	0.569	0.930	0.725	0.985	0.878	0.962	0.970	0.622	0.937	0.993	0.854	**0.046***	0.986	0.913
PCaaC420	0.949	0.930	0.765	0.985	0.937	0.962	0.970	0.829	0.892	0.993	0.854	0.166	0.986	0.913
PCaaC421	0.804	0.930	0.541	0.985	0.878	0.999	0.970	0.554	0.892	0.993	0.854	**0.016***	0.986	0.994
PCaaC422	0.676	0.930	0.477	0.985	0.916	0.999	0.970	0.242	0.892	0.993	0.962	**0.010***	0.986	0.913
PCaaC424	0.807	0.930	0.477	0.985	0.894	0.999	0.975	0.727	0.892	0.993	0.907	0.088	0.986	0.977
PCaaC425	0.610	0.930	0.776	0.996	0.878	0.962	0.975	0.749	0.892	0.993	0.877	0.286	0.986	0.635
PCaaC426	0.844	0.930	0.792	0.985	0.927	0.999	0.970	0.464	0.892	0.993	0.993	**0.028***	0.986	0.913
PCaeC300	0.426	0.930	0.569	0.985	0.880	0.962	0.970	0.692	0.924	0.993	0.907	0.401	0.986	0.414
PCaeC301	0.580	0.930	0.477	0.985	0.878	0.962	0.970	0.242	0.892	0.993	0.976	**0.010***	0.986	0.794

PCaeC302	0.769	0.930	0.569	0.985	0.963	0.999	0.980	0.315	0.892	0.993	0.907	0.059	0.986	0.552
PCaeC321	0.439	0.930	0.569	0.996	0.878	0.962	0.970	0.554	0.789	0.993	0.907	**0.035***	0.986	0.913
PCaeC322	0.804	0.930	0.479	0.985	0.927	0.975	0.970	0.778	0.892	0.993	0.854	**0.024***	0.986	0.913
PCaeC340	0.610	0.930	0.665	0.985	0.878	0.878	0.975	0.554	0.887	0.993	0.893	**0.020***	0.986	0.913
PCaeC341	0.610	0.966	0.569	0.985	0.878	0.962	0.970	0.554	0.976	0.993	0.893	0.052	0.986	0.913
PCaeC342	0.536	0.930	0.569	0.985	0.878	0.962	0.970	0.554	0.950	0.993	0.854	**0.030***	0.986	0.913
PCaeC343	0.528	0.930	0.587	0.985	0.878	0.962	0.970	0.560	0.937	0.993	0.854	**0.035***	0.986	0.913
PCaeC360	0.667	0.930	0.477	0.985	0.878	0.962	0.970	0.385	0.892	0.993	0.907	**0.010***	0.986	0.913
PCaeC361	0.610	0.934	0.477	0.985	0.878	0.962	0.970	0.554	0.892	0.993	0.854	**0.020***	0.986	0.913
PCaeC362	0.510	0.930	0.477	0.985	0.878	0.962	0.970	0.554	0.892	0.993	0.854	**0.016***	0.986	0.913
PCaeC363	0.610	0.977	0.477	0.987	0.878	0.962	0.970	0.632	0.892	0.993	0.854	0.081	0.986	0.913
PCaeC364	0.536	0.930	0.569	0.985	0.878	0.962	0.970	0.554	0.892	0.993	0.854	**0.022***	0.986	0.913
PCaeC365	0.426	0.930	0.569	0.985	0.937	0.999	0.970	0.554	0.924	0.993	0.854	**0.022***	0.987	0.913
PCaeC380	0.777	0.930	0.477	0.996	0.878	0.962	0.970	0.554	0.892	0.993	0.854	0.075	0.986	0.913
PCaeC381	0.777	0.930	0.632	0.985	0.878	0.962	0.980	0.554	0.887	0.993	0.854	**0.030***	0.986	0.954
PCaeC382	0.536	0.930	0.662	0.985	0.878	0.962	0.970	0.692	0.892	0.993	0.854	0.103	0.986	0.968
PCaeC383	0.528	0.930	0.541	0.985	0.927	0.975	0.970	0.464	0.892	0.993	0.854	**0.016***	0.986	0.913
PCaeC384	0.725	0.930	0.477	0.985	0.927	0.966	0.970	0.554	0.892	0.993	0.854	**0.040***	0.986	0.913
PCaeC385	0.454	0.930	0.596	0.985	0.878	0.962	0.970	0.554	0.892	0.993	0.854	**0.024***	0.986	0.913
PCaeC386	0.644	0.930	0.651	0.985	0.878	0.962	0.970	0.555	0.892	0.993	0.893	**0.030***	0.986	0.986
PCaeC401	0.619	0.998	0.477	0.985	0.927	0.962	0.970	0.469	0.887	0.993	0.907	**0.022***	0.986	0.977
PCaeC402	0.949	0.930	0.569	0.985	0.878	0.962	0.970	0.632	0.892	0.993	0.907	**0.033***	0.986	0.977
PCaeC403	0.497	0.930	0.477	0.985	0.878	0.934	0.970	0.554	0.924	0.993	0.907	**0.024***	0.986	0.913
PCaeC404	0.542	0.930	0.578	0.996	0.927	0.962	0.975	0.554	0.892	0.993	0.854	**0.010***	0.986	0.913
PCaeC405	0.542	0.930	0.477	0.985	0.878	0.962	0.970	0.554	0.892	0.993	0.854	**0.035***	0.986	0.913
PCaeC406	0.454	0.930	0.477	0.985	0.904	0.962	0.970	0.554	0.892	0.993	0.854	**0.028***	0.986	0.913
PCaeC421	0.569	0.930	0.569	0.985	0.908	0.962	0.970	0.871	0.892	0.993	0.854	**0.030***	0.986	0.913
PCaeC422	0.375	0.930	0.477	0.985	0.878	0.962	0.970	0.554	0.892	0.993	0.976	0.129	0.986	0.986
PCaeC423	0.725	0.930	0.477	0.985	0.878	0.999	0.980	0.554	0.892	0.993	0.854	**0.010***	0.986	0.913
PCaeC424	0.426	0.977	0.662	0.985	0.937	0.962	0.970	0.554	0.887	0.993	0.854	0.286	0.986	0.913
PCaeC425	0.528	0.930	0.477	0.985	0.878	0.999	0.970	0.555	0.892	0.993	0.854	**0.020***	0.986	0.994
PCaeC443	0.539	0.930	0.651	0.985	0.878	0.962	0.970	0.727	0.892	0.993	0.993	0.979	0.986	0.913
PCaeC444	0.640	0.930	0.569	0.996	0.878	0.999	0.970	0.385	0.789	0.993	0.854	0.064	0.986	0.913
PCaeC445	0.520	0.930	0.477	0.989	0.878	0.962	0.970	0.554	0.892	0.993	0.854	**0.026***	0.986	0.913

PCaeC446	0.426	0.977	0.569	0.985	0.878	0.962	0.975	0.692	0.887	0.993	0.854	**0.024***	0.986	0.913
SMOHC141	0.138	0.800	0.877	0.985	0.878	0.962	0.970	0.622	0.789	0.993	0.854	**0.010***	0.986	0.913
SMOHC161	0.399	0.930	0.838	0.985	0.937	0.977	0.970	0.794	0.892	0.993	0.907	**0.036***	0.986	0.913
SMOHC221	0.729	0.930	0.981	0.985	0.878	0.962	0.970	0.991	0.892	0.993	0.907	0.590	0.986	0.913
SMOHC222	0.610	0.966	0.810	0.985	0.878	0.977	0.970	0.764	0.892	0.993	0.907	0.755	0.986	0.913
SMC160	0.138	0.800	0.925	0.985	0.878	0.962	0.970	0.827	0.789	0.993	0.894	**0.028***	0.986	0.977
SMC161	0.138	0.800	0.736	0.985	0.985	0.962	0.975	0.554	0.887	0.993	0.962	**0.035***	0.986	0.913
SMC180	0.293	0.930	0.925	0.985	0.985	0.999	0.970	0.692	0.789	0.993	0.854	**0.020***	0.986	0.977
SMC181	0.398	0.930	0.993	0.985	0.927	0.962	0.970	0.554	0.789	0.993	0.854	**0.010***	0.986	0.954
SMC240	0.398	0.930	0.736	0.985	0.878	0.962	0.995	0.685	0.887	0.993	0.907	0.125	0.986	0.913
H1	0.375	0.930	0.477	0.985	0.937	0.962	0.970	0.685	0.887	0.993	0.854	0.391	0.986	0.973

Metabolites	Painkiller	Antibiotics				Others							
	Analgesics (Yes/No)	Fluclox (Yes/No)	Cefuroxime (Yes/No)	Teicoplanin (Yes/No)	Gentamicin (Yes/No)	Hypoten-sion (Yes/No)	Ephedrine (Yes/No)	Tachycardia (Yes/No)	Dexame-tha-sone (Yes/No)	Magnesium Sulphate (Yes/No)	Ono-danosetro no (Yes/No)	Chlor-phe-nira-mine (Yes/No)	prochlorpera-zine(Yes/No)
	q-Value	q-Value	q-Value	q-Value	q-Value	q-Value	q-Value	q-Value	q-Value	q-Value	q-Value	q-Value	q-Value
C0	0.802	1.000	0.998	0.967	0.994	0.995	0.754	0.296	0.999	0.946	0.993	0.946	0.996
C141	0.802	1.000	0.998	0.967	0.994	0.995	0.783	0.986	0.999	0.946	0.993	0.946	0.996
C142	0.802	1.000	0.998	0.967	0.994	0.995	0.754	0.134	0.999	0.946	0.993	0.946	0.996
C16	0.802	1.000	0.998	0.967	0.994	0.995	0.888	0.120	0.999	0.946	0.993	0.968	0.996
C161	0.802	1.000	0.998	0.967	0.994	0.995	0.754	0.528	0.999	0.946	0.993	0.946	0.996
C18	0.802	1.000	0.998	0.967	0.994	0.999	0.811	0.851	0.999	0.974	0.993	0.946	0.996
C181	0.802	1.000	0.998	0.977	0.994	0.995	0.992	0.263	0.999	0.952	0.993	0.968	0.996
C2	0.802	1.000	0.998	0.967	0.994	0.995	0.754	0.499	0.999	0.946	0.993	0.968	0.996
C3	0.802	1.000	0.998	0.967	0.994	0.995	0.754	0.372	0.999	0.946	0.993	0.946	0.996
C5	0.806	1.000	0.998	0.967	0.994	0.995	0.808	0.356	0.999	0.946	0.993	0.946	0.996
C51DC	0.802	1.000	0.998	0.414	0.994	0.995	0.754	0.622	0.999	0.946	0.993	0.946	0.996
C7DC	0.996	1.000	0.998	0.967	0.994	0.995	0.783	0.113	0.999	0.946	0.993	0.961	0.996
Ala	0.802	1.000	0.998	0.967	0.994	0.995	0.754	0.298	0.999	0.946	0.993	0.946	0.996
Arg	0.850	1.000	0.998	0.967	0.994	0.995	0.754	0.134	0.999	0.946	0.993	0.800	0.996
Asn	0.996	1.000	0.998	0.967	0.994	0.995	0.754	0.365	0.999	0.946	0.993	0.800	0.996
Asp	0.802	1.000	0.998	0.998	0.994	0.995	0.754	0.356	0.999	0.946	0.993	0.946	0.996
Cit	0.802	1.000	0.998	0.967	0.994	0.995	0.754	0.581	0.999	0.946	0.993	0.946	0.996
Gln	0.996	1.000	0.998	0.967	0.994	0.995	0.868	0.134	0.999	0.952	0.993	0.946	0.996
Glu	0.802	1.000	0.998	0.967	**0.028***	0.995	0.754	0.810	0.999	0.946	0.993	0.946	0.996
Gly	0.802	1.000	0.998	0.967	0.994	0.995	0.857	0.302	0.999	0.946	0.993	0.963	0.996
His	0.900	1.000	0.998	0.967	0.994	0.995	0.822	0.208	0.999	0.946	0.993	0.946	0.996
Ile	0.996	1.000	0.998	0.967	0.994	0.995	0.754	0.450	0.999	0.946	0.993	0.552	0.996
Leu	0.802	1.000	0.998	0.967	0.994	0.995	0.934	0.554	0.999	0.946	0.993	0.946	0.996
Lys	0.802	1.000	0.998	0.967	0.994	0.995	0.754	0.292	0.999	0.946	0.993	0.946	0.996
Met	0.802	1.000	0.998	0.967	0.994	0.995	0.754	0.107	0.999	0.946	0.993	0.800	0.996
Orn	0.802	1.000	0.998	0.967	0.994	0.995	0.947	0.134	0.999	0.946	0.993	0.946	0.996
Phe	0.996	1.000	0.998	0.967	0.994	0.995	0.754	0.134	0.999	0.946	0.993	0.946	0.996
Pro	0.806	1.000	0.998	0.967	0.994	0.995	0.898	0.499	0.999	0.946	0.993	0.946	0.996

Ser	0.996	1.000	0.998	0.967	0.994	0.995	0.783	0.120	0.999	0.952	0.993	0.946	0.996
Thr	0.802	1.000	0.998	0.967	0.994	0.995	0.754	0.134	0.999	0.946	0.993	0.946	0.996
Trp	0.802	1.000	0.998	0.967	0.994	0.995	0.772	0.136	0.999	0.946	0.993	0.949	0.996
Tyr	0.996	1.000	0.998	0.967	0.994	0.995	0.754	0.188	0.999	0.946	0.993	0.946	0.996
Val	0.996	1.000	0.998	0.967	0.994	0.995	0.754	0.102	0.999	0.946	0.993	0.946	0.996
ADMA	0.802	1.000	0.998	0.967	0.994	0.995	0.754	0.581	0.999	0.946	0.993	0.946	0.996
Creatine	0.820	1.000	0.998	0.977	0.994	0.995	0.992	0.851	0.999	0.946	0.993	0.946	0.996
Kynurenine	0.802	1.000	0.998	0.967	0.994	0.995	0.916	0.102	0.999	0.946	0.993	0.949	0.996
MetSO	0.802	1.000	0.998	0.967	0.994	0.995	0.772	0.939	0.999	0.974	0.993	0.968	0.996
Putrescine	0.875	1.000	0.998	0.977	0.994	0.995	0.920	0.255	0.999	0.946	0.993	0.946	0.996
Sarcosine	0.996	1.000	0.998	0.967	0.994	0.995	0.783	0.763	0.999	0.946	0.993	0.946	0.996
Serotonin	0.933	1.000	0.998	0.967	0.994	0.999	0.783	0.933	0.999	0.946	0.993	0.946	0.996
Spermidine	0.802	1.000	0.998	0.967	0.994	0.995	0.754	0.248	0.999	0.946	0.993	0.946	0.996
Spermine	0.802	1.000	0.998	0.967	0.994	0.995	0.981	0.581	0.999	0.946	0.993	0.946	0.996
t4OHPro	0.802	1.000	0.998	0.977	0.552	0.995	0.903	0.520	0.999	0.946	0.993	0.946	0.996
Taurine	0.821	1.000	0.998	0.967	0.897	0.995	0.754	0.148	0.999	0.946	0.993	0.946	0.996
SDMA	0.806	1.000	0.998	0.967	0.994	0.995	0.790	0.302	0.999	0.946	0.993	0.963	0.996
lysoPCaC160	0.802	1.000	0.998	0.967	0.994	0.995	0.783	0.134	0.999	0.985	0.993	0.946	0.996
lysoPCaC161	0.802	1.000	0.998	0.967	0.994	0.995	0.754	0.302	0.999	0.946	0.993	0.946	0.996
lysoPCaC170	0.802	1.000	0.998	0.967	0.994	0.995	0.754	0.134	0.999	0.946	0.993	0.946	0.996
lysoPCaC180	0.802	1.000	0.998	0.967	0.994	0.995	0.811	0.134	0.999	0.974	0.993	0.946	0.996
lysoPCaC181	0.802	1.000	0.998	0.967	0.994	0.995	0.754	0.140	0.999	0.946	0.993	0.949	0.996
lysoPCaC182	0.808	1.000	0.998	0.967	0.994	0.995	0.754	0.134	0.999	0.946	0.993	0.946	0.996
lysoPCaC203	0.968	1.000	0.998	0.967	0.994	0.995	0.754	0.102	0.999	0.952	0.993	0.946	0.996
lysoPCaC204	0.802	1.000	0.998	0.967	0.994	0.995	0.754	0.298	0.999	0.946	0.993	0.968	0.996
lysoPCaC240	0.802	1.000	0.998	0.973	0.994	0.995	0.754	0.813	0.999	0.952	0.993	0.968	0.996
lysoPCaC260	0.802	1.000	0.998	0.967	0.994	0.995	0.754	0.520	0.999	0.946	0.993	0.946	0.996
lysoPCaC261	0.802	1.000	0.998	0.967	0.994	0.995	0.754	0.381	0.999	0.946	0.993	0.946	0.996
lysoPCaC280	0.802	1.000	0.998	0.967	0.994	0.995	0.754	0.965	0.999	0.946	0.993	0.946	0.996
lysoPCaC281	0.802	1.000	0.998	0.967	0.994	0.995	0.754	0.292	0.999	0.946	0.993	0.946	0.996
PCaaC240	0.802	1.000	0.998	0.967	0.994	0.995	0.754	0.838	0.999	0.946	0.993	0.946	0.996
PCaaC281	0.802	1.000	0.998	0.967	0.994	0.995	0.754	0.102	0.999	0.946	0.993	0.946	0.996
PCaaC300	0.802	1.000	0.998	0.967	0.994	0.995	0.754	0.146	0.999	0.946	0.993	0.946	0.996
PCaaC320	0.802	1.000	0.998	0.967	0.994	0.995	0.754	0.179	0.999	0.946	0.993	0.949	0.996

PCaaC321	0.802	1.000	0.998	0.967	0.994	0.999	0.754	0.102	0.999	0.946	0.993	0.963	0.996
PCaaC323	0.802	1.000	0.998	0.967	0.994	0.995	0.754	0.188	0.999	0.946	0.993	0.968	0.996
PCaaC341	0.802	1.000	0.998	0.967	0.994	0.995	0.772	0.114	0.999	0.946	0.993	0.968	0.996
PCaaC342	0.802	1.000	0.998	0.967	0.994	0.995	0.754	0.102	0.999	0.946	0.993	0.946	0.996
PCaaC343	0.802	1.000	0.998	0.967	0.994	0.995	0.783	0.107	0.999	0.946	0.993	0.968	0.996
PCaaC344	0.802	1.000	0.998	0.967	0.994	0.995	0.783	0.131	0.999	0.946	0.993	0.963	0.996
PCaaC360	0.802	1.000	0.998	0.967	0.994	0.995	0.979	0.302	0.999	0.946	0.690	0.946	0.996
PCaaC361	0.802	1.000	0.998	0.967	0.994	0.995	0.772	0.114	0.999	0.946	0.993	0.963	0.996
PCaaC362	0.802	1.000	0.998	0.967	0.994	0.995	0.754	0.102	0.999	0.946	0.993	0.946	0.996
PCaaC363	0.802	1.000	0.998	0.967	0.994	0.995	0.754	0.102	0.999	0.946	0.993	0.946	0.996
PCaaC364	0.802	1.000	0.998	0.967	0.994	0.995	0.772	0.102	0.999	0.946	0.993	0.946	0.996
PCaaC365	0.821	1.000	0.998	0.967	0.994	0.995	0.754	0.102	0.999	0.946	0.993	0.946	0.996
PCaaC366	0.948	1.000	0.998	0.967	0.994	0.995	0.772	0.212	0.999	0.946	0.993	0.946	0.996
PCaaC380	0.802	1.000	0.998	0.967	0.994	0.995	0.988	0.134	0.999	0.946	0.993	0.968	0.996
PCaaC383	0.802	1.000	0.998	0.998	0.994	0.995	0.754	0.102	0.999	0.946	0.993	0.946	0.996
PCaaC384	0.821	1.000	0.998	0.967	0.994	0.995	0.754	0.102	0.999	0.946	0.993	0.946	0.997
PCaaC385	0.802	1.000	0.998	0.977	0.994	0.995	0.783	0.108	0.999	0.946	0.993	0.946	0.996
PCaaC386	0.868	1.000	0.998	0.967	0.994	0.995	0.754	0.102	0.999	0.946	0.993	0.946	0.996
PCaaC402	0.802	1.000	0.998	0.967	0.994	0.995	0.754	0.851	0.999	0.946	0.993	0.946	0.996
PCaaC403	0.802	1.000	0.998	0.967	0.994	0.995	0.783	0.263	0.999	0.946	0.993	0.946	0.996
PCaaC404	0.996	1.000	0.998	0.977	0.994	0.995	0.754	0.134	0.999	0.946	0.993	0.946	0.996
PCaaC405	0.802	1.000	0.998	0.977	0.994	0.995	0.808	0.134	0.999	0.946	0.993	0.963	0.996
PCaaC406	0.802	1.000	0.998	0.967	0.994	0.995	0.754	0.102	0.999	0.946	0.993	0.946	0.996
PCaaC420	0.996	1.000	0.998	0.967	0.994	0.995	0.903	0.105	0.999	0.946	0.993	0.946	0.996
PCaaC421	0.875	1.000	0.998	0.967	0.994	0.995	0.908	0.581	0.999	0.946	0.993	0.946	0.996
PCaaC422	0.802	1.000	0.998	0.967	0.994	0.995	0.783	0.528	0.999	0.946	0.993	0.946	0.996
PCaaC424	0.802	1.000	0.998	0.967	0.994	0.995	0.997	0.686	0.999	0.946	0.993	0.972	0.996
PCaaC425	0.996	1.000	0.998	0.973	0.994	0.995	0.771	0.340	0.999	0.946	0.993	0.946	0.996
PCaaC426	0.968	1.000	0.998	0.967	0.994	0.995	0.754	0.298	0.999	0.946	0.993	0.973	0.996
PCaeC300	0.802	1.000	0.998	0.967	0.994	0.995	0.754	0.146	0.999	0.946	0.993	0.946	0.996
PCaeC301	0.802	1.000	0.998	0.967	0.994	0.995	0.772	0.248	0.999	0.952	0.993	0.968	0.996
PCaeC302	0.802	1.000	0.998	0.967	0.994	0.995	0.754	0.334	0.999	0.946	0.993	0.946	0.996
PCaeC321	0.802	1.000	0.998	0.967	0.994	0.995	0.754	0.148	0.999	0.946	0.993	0.946	0.996
PCaeC322	0.802	1.000	0.998	0.973	0.994	0.995	0.771	0.175	0.999	0.946	0.993	0.946	0.996

PCaeC340	0.802	1.000	0.998	0.967	0.994	0.995	0.754	0.102	0.999	0.946	0.993	0.981	0.996
PCaeC341	0.802	1.000	0.998	0.967	0.994	0.995	0.754	0.134	0.999	0.946	0.993	0.946	0.996
PCaeC342	0.873	1.000	0.998	0.967	0.994	0.995	0.754	0.102	0.999	0.946	0.993	0.946	0.996
PCaeC343	0.996	1.000	0.998	0.967	0.994	0.995	0.754	0.120	0.999	0.946	0.993	0.946	0.996
PCaeC360	0.996	1.000	0.998	0.977	0.994	0.995	0.754	0.292	0.999	0.946	0.993	0.946	0.996
PCaeC361	0.968	1.000	0.998	0.967	0.994	0.999	0.754	0.102	0.999	0.946	0.993	0.946	0.996
PCaeC362	0.802	1.000	0.998	0.967	0.994	0.995	0.754	0.120	0.999	0.985	0.993	0.946	0.996
PCaeC363	0.996	1.000	0.998	0.973	0.994	0.995	0.754	0.134	0.999	0.946	0.993	0.946	0.996
PCaeC364	0.939	1.000	0.998	0.967	0.994	0.995	0.754	0.102	0.999	0.946	0.993	0.946	0.996
PCaeC365	0.996	1.000	0.998	0.967	0.994	0.995	0.754	0.102	0.999	0.946	0.993	0.946	0.996
PCaeC380	0.802	1.000	0.998	0.967	0.994	0.995	0.988	0.296	0.999	0.946	0.993	0.949	0.996
PCaeC381	0.996	1.000	0.998	0.967	0.994	0.995	0.754	0.188	0.999	0.946	0.993	0.946	0.996
PCaeC382	0.982	1.000	0.998	0.967	0.994	0.995	0.754	0.263	0.999	0.946	0.993	0.800	0.996
PCaeC383	0.996	1.000	0.998	0.967	0.994	0.995	0.754	0.134	0.999	0.946	0.993	0.946	0.996
PCaeC384	0.996	1.000	0.998	0.967	0.994	0.995	0.754	0.134	0.999	0.946	0.993	0.946	0.996
PCaeC385	0.996	1.000	0.998	0.967	0.994	0.995	0.754	0.102	0.999	0.946	0.993	0.946	0.996
PCaeC386	0.900	1.000	0.998	0.967	0.994	0.995	0.754	0.102	0.999	0.946	0.993	0.946	0.996
PCaeC401	0.802	1.000	0.998	0.997	0.994	0.995	0.754	0.302	0.999	0.946	0.993	0.946	0.996
PCaeC402	0.802	1.000	0.998	0.967	0.994	0.995	0.754	0.513	0.999	0.946	0.993	0.946	0.996
PCaeC403	0.802	1.000	0.998	0.967	0.994	0.995	0.754	0.231	0.999	0.946	0.993	0.946	0.996
PCaeC404	0.802	1.000	0.998	0.967	0.994	0.995	0.754	0.102	0.999	0.946	0.993	0.946	0.996
PCaeC405	0.968	1.000	0.998	0.967	0.994	0.995	0.754	0.134	0.999	0.946	0.993	0.946	0.996
PCaeC406	0.996	1.000	0.998	0.967	0.994	0.995	0.754	0.102	0.999	0.946	0.993	0.946	0.996
PCaeC421	0.948	1.000	0.998	0.967	0.994	0.995	0.772	0.450	0.999	0.946	0.993	0.946	0.996
PCaeC422	0.802	1.000	0.998	0.967	0.994	0.995	0.754	0.851	0.999	0.946	0.993	0.946	0.996
PCaeC423	0.948	1.000	0.998	0.967	0.994	0.995	0.754	0.292	0.999	0.946	0.993	0.946	0.996
PCaeC424	0.802	1.000	0.998	0.967	0.994	0.995	0.754	0.134	0.999	0.946	0.993	0.968	0.996
PCaeC425	0.968	1.000	0.998	0.967	0.994	0.995	0.754	0.134	0.999	0.946	0.993	0.946	0.996
PCaeC443	0.868	1.000	0.998	0.967	0.994	0.995	0.771	0.986	0.999	0.946	0.993	0.946	0.996
PCaeC444	0.996	1.000	0.998	0.967	0.994	0.995	0.790	0.866	0.999	0.946	0.993	0.993	0.996
PCaeC445	0.996	1.000	0.998	0.967	0.994	0.995	0.772	0.102	0.999	0.946	0.993	0.946	0.996
PCaeC446	0.802	1.000	0.998	0.967	0.994	0.995	0.754	0.102	0.999	0.946	0.993	0.946	0.996
SMOHC141	0.968	1.000	0.998	0.967	0.994	0.995	0.754	0.102	0.999	0.946	0.993	0.946	0.996
SMOHC161	0.802	1.000	0.998	0.967	0.994	0.995	0.754	0.292	0.999	0.974	0.993	0.946	0.996

SMOHC221	0.996	1.000	0.998	0.967	0.414	0.995	0.916	0.265	0.999	0.946	0.993	0.946	0.996
SMOHC222	0.802	1.000	0.998	0.967	0.994	0.995	0.934	0.579	0.999	0.946	0.993	0.963	0.996
SMC160	0.802	1.000	0.998	0.967	0.994	0.995	0.772	0.102	0.999	0.946	0.993	0.946	0.996
SMC161	0.864	1.000	0.998	0.977	0.994	0.995	0.772	0.105	0.999	0.946	0.993	0.946	0.996
SMC180	0.812	1.000	0.998	0.977	0.994	0.995	0.868	0.134	0.999	0.946	0.993	0.946	0.996
SMC181	0.802	1.000	0.998	0.967	0.994	0.995	0.947	0.134	0.999	0.946	0.993	0.946	0.996
SMC240	0.802	1.000	0.998	0.977	0.994	0.995	0.783	0.134	0.999	0.946	0.993	0.946	0.996
H1	0.802	1.000	0.998	0.967	0.994	0.995	0.908	0.538	0.999	0.946	0.993	0.946	0.996

q-values are from Benjamini-Hochberg, and significant q-values are in bold and present *q < 0.05, **q < 0.01 perioperative clinical factors vs. % change in metabolites. Multi-premedication includes ranitidine, PPI, and benzodiazepine. PPI; Proton-pump inhibitor; Ala: alanine; Arg: arginine; Asn: asparagine; Asp: aspartate; Cit: citrulline ; Gln: glutamine; Glu: glutamate; Gly: glycine; His: histidine; Ile: isoleucine; Leu: leucine; Lys: lysine; Met: methionine; Orn: ornithine; Phe: phenylalanine; Pro: proline; Ser: serine; Thr: threonine; Trp: tryptophan; Tyr: tyrosine; Val: valine; ADMA: asymmetric dimethylarginine; SDMA:symmetric dimethylarginine; H1: hexose.

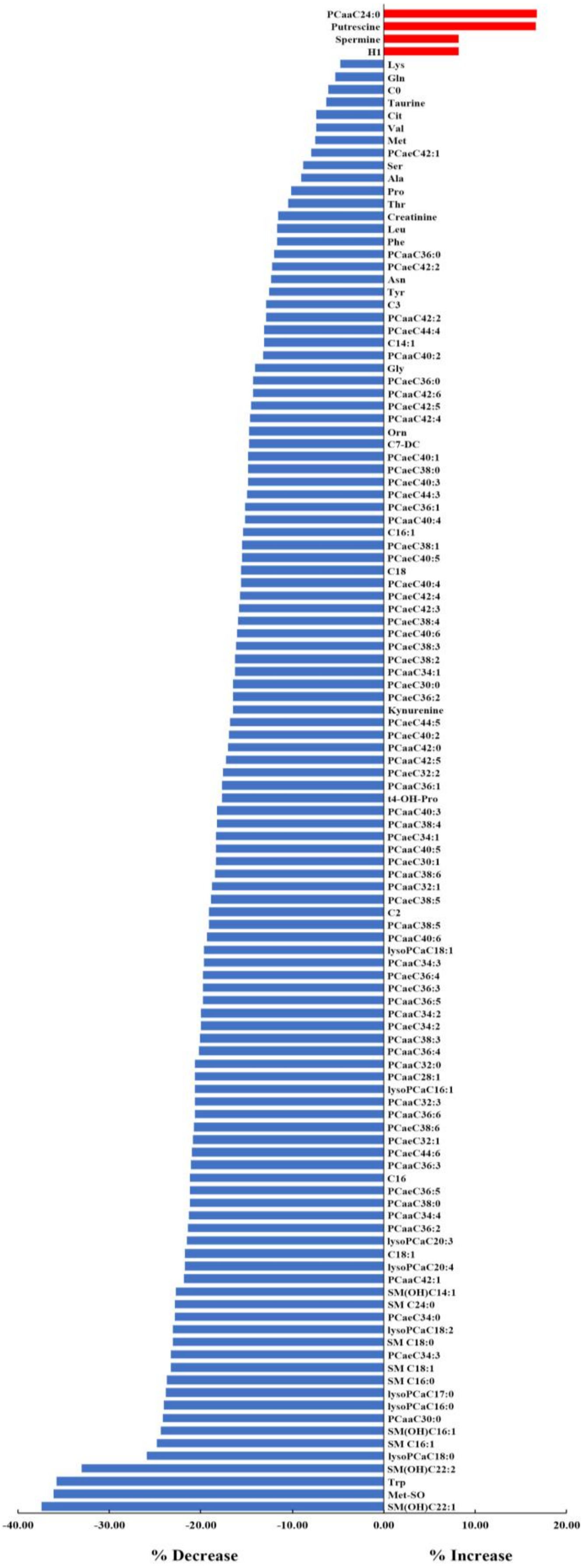

Supplementary Figure S1. The % change rate of 121 metabolites between pre- and post-op.
